# Establishing Cardiac MRI Reference Ranges Stratified by Sex and Age for Cardiovascular function during Exercise

**DOI:** 10.1148/ryct.240175

**Published:** 2025-06-01

**Authors:** Ronny Schweitzer, Antonio de Marvao, Mit Shah, Paolo Inglese, Peter Kellman, Alaine Berry, Ben Statton, Declan P O’Regan

**Affiliations:** 1 MRC Laboratory of Medical Sciences, Imperial College London, Hammersmith Hospital Campus, London, UK; 2 Department of Women and Children’s Health, King’s College London, London, UK; 3 British Heart Foundation Centre of Research Excellence, School of Cardiovascular Medicine and Sciences, King’s College London, London, UK; 4 National Heart, Lung, and Blood Institute, National Institutes of Health, Bethesda, MD, USA

## Abstract

**Purpose:**

To evaluate the effects of exercise on left ventricular parameters using exercise cardiac MRI in healthy adults without known cardiovascular disease, and establish reference ranges stratified by age and sex.

**Materials and Methods:**

This prospective study included healthy adult participants with no known cardiovascular disease or genetic variants associated with cardiomyopathy, enrolled between January 2018 and April 2021, who underwent exercise cardiac MRI evaluation. Participants were imaged at rest and after exercise, with parameters measured by two readers. Prediction intervals were calculated and compared across sex and age groups.

**Results:**

The study included 161 participants (mean age, 49±[SD]14 years; 85 female). Compared with the resting state, exercise caused an increase in heart rate (64±9 bpm vs 133±19 bpm, P < 0.001), left ventricular end-diastolic volume (140±32 ml vs 148±35 ml, P < 0.001), stroke volume (82±18 ml vs 102±25 ml, P < 0.001), ejection fraction (59±6% vs 69±7%, P < 0.001), and cardiac output (5.2±1.1 l/min vs 13.5±3.9 l/min, P < 0.001), and a decrease in left ventricular end-systolic volume (58±18 ml vs 46±15 ml, P < 0.001). There were significant differences in exercise response between groups stratified by sex and age for most parameters.

**Conclusion:**

In healthy adults, an increase in cardiac output after exercise is driven by a rise in heart rate with both increased ventricular filling and emptying. Normal ranges for exercise response, stratified by age and sex, are established as a reference for the use of exercise cardiac MRI in clinical practice.

## Introduction

Exercise cardiac MRI is a non-invasive imaging modality that offers high image quality during physiological stress. It can be undertaken as part of a comprehensive cardiac MRI study of structure, function and tissue characterization to assess physiological reserve during maximal exercise. This is typically performed using real-time cine imaging, as well as other cardiac MRI sequences to assess myocardial strain, aortic flow or tissue metabolism. The technique has emerging applications across several conditions, including the stratification of patients with pulmonary hypertension, diagnosing athlete’s heart, evaluating exercise tolerance in congenital heart disease, and detecting ischemia.^1^ The use of exercise cardiac MRI offers advantages over exercise echocardiography due to its reliable assessment of systolic and diastolic function, which is less user-dependent.^2^ Use of continuous electrocardiogram-triggered real-time (RT) imaging eliminates the requirement for breath-holding, which may itself affect cardiac filling pressures.^3^ Physical exercise has a higher diagnostic sensitivity and fewer adverse events compared to pharmacological stressors and is recommended for stress imaging whenever feasible.^4^

Knowledge of the range of normal function in exercise is needed for clinical interpretation,^5^ but no studies to date have reported reference ranges for exercise cardiac MRI. With the widening use of exercise cardiac MRI in both clinical and research domains, there is a need to develop representative values in healthy individuals. It has been established that exercise cardiac MRI is clinically feasible using commercially available exercise equipment and vendor-provided product sequences.^6^ The purpose of this study is to evaluate the effects of exercise on left ventricular parameters using exercise cardiac MRI in a cohort of healthy adults without known cardiovascular disease or major comorbidities, and to establish reference ranges stratified by age and sex.

## Materials and Methods

### Study Design and Sample

This was a prospective, observational, single center substudy performed at Imperial College London. The overall study aimed to evaluate the role of genetic variation on cardiovascular function in a clinically healthy cohort using cardiac MRI. The current study includes a subcohort of participants in which exercise cardiac MRI was used to assess functional reserve. This study received research ethics approval from the West London Research Ethics Committee (17/LO/0034) and all participants provided written informed consent prior to study enrollment.

Enrollment was between January 2018 and April 2021. Individuals were randomly selected from a cohort of almost 2000 healthy volunteers that had been recruited by advertisement.^7^ Participants were eligible for inclusion if they were over the age of 18 years, had no history of cardiovascular disease, and were genotype-negative for inherited cardiac conditions during genetic screening. Exclusion criteria included known or suspected pregnancy, physical limitations that would preclude participants from exercising, and any contraindications to MRI. All participants completed a health questionnaire and underwent assessment of height and weight, blood pressure, and a resting 12-lead electrocardiogram.

### Image Acquisition

**Figure 1:**
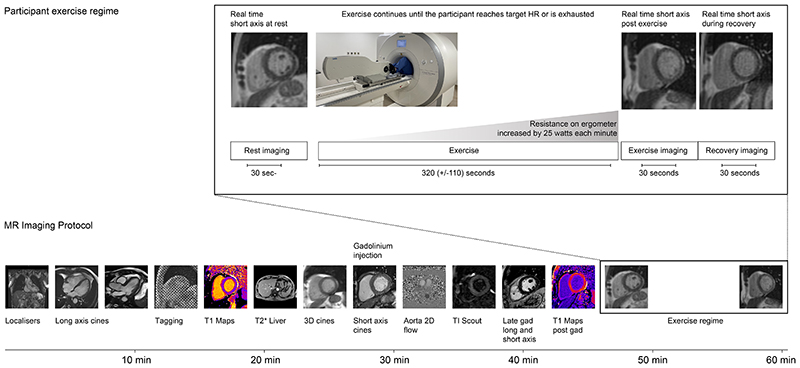
Imaging protocol. Exercise regime and imaging protocol used in the study. HR = heart rate

Images were acquired using a 1.5T MAGNETOM Aera (Siemens Healthineers, Erlangen, Germany) using a 60 channel cardiac coil. Participants underwent a complete cardiac MRI protocol including structural and functional imaging, phase-contrast flow assessment and late-gadolinium enhancement (Figure 1) following published guidelines and standards.^8^ Following this, short axis cines were acquired at rest and exercise using a commercially available conventional RT balanced steady-state free precession sequence. The short-axis slices provided complete coverage from base to apex, allowing for variation in heart size and movement during exercise. Each slice was triggered on the electrocardiogram R-wave and then acquired free-running for 3 seconds per slice. As images were acquired over multiple heart beats, this enabled a retrospective reconstruction to a pre-defined number of cardiac phases to simplify subsequent volumetric analysis. Here the output images were reconstructed at the required times using a 5th order B-spline interpolation of the raw data in the time domain on a pixelwise basis. This approach introduces no bias to volumetric assessment compared to conventional reconstruction.^9^ Typical acquisition parameters were: repetition time = 2.2 ms, echo time = 0.97 ms, slice thickness = 8 mm, slice gap = 2 mm, matrix size = 128 × 96, field of view = 360 x 270 mm. The acquisition was accelerated using a phase resolution of 75%, partial Fourier 6/8 and a GeneRalized Autocalibrating Partial Parallel Acquisition (GRAPPA) factor = 4 with a temporal parallel acquisition reference scan technique. This achieved a temporal resolution of 31 ms.

### Exercise Protocol

**Figure 2:**
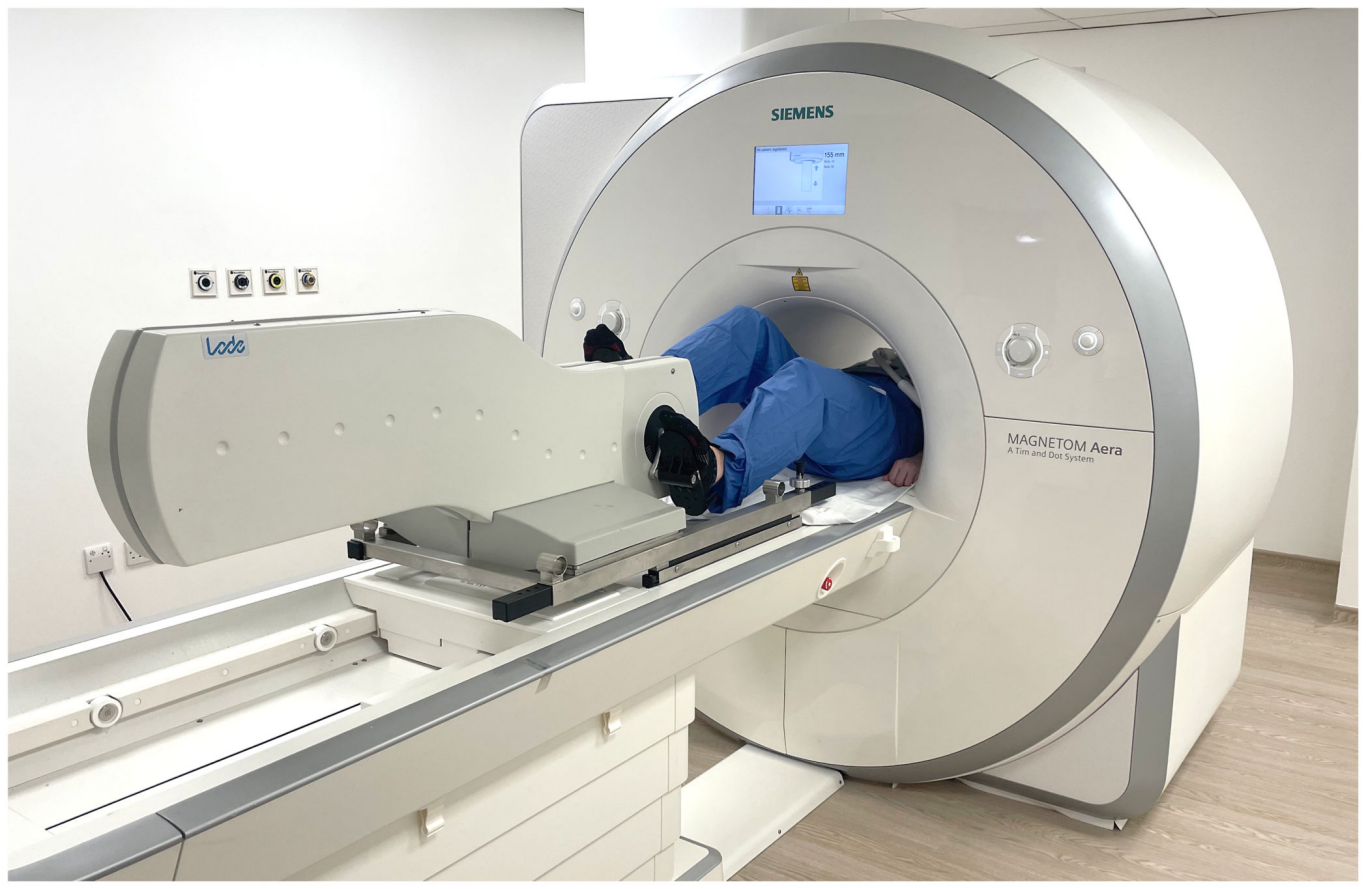
Pedal ergometer. Positioning of the bed-mounted MRI-conditional pedal ergometer allowing participants to exercise while at isocentre (hand holds not shown). An electro-magnetic braking system allows a maximum peak load of up to 300W. An external control unit displays multiple ergometry parameters in real time.

Participants were instructed to abstain from eating but to drink freely in the 6 hours prior to their study. Exercise was performed using a MRI-conditional variable resistance supine ergometer (Lode BV, Netherlands; Figure 2, Video S1) attached to the scanner table. Participants were asked to grip the handles of the ergometer and coached to keep their torso as stationary as possible during exercise to minimize bulk movement. The exercise protocol was based on a proposed framework for cardiopulmonary exercise testing,^10^ which is similar to approaches taken for supine exercise cardiac MRI at other centers.^1^ The target heart rate (HR) for each participant was calculated as 85% of the maximal HR using Fox’s formula (220 - age in years).^11^

Following resting RT imaging, the participants were instructed to start exercising while maintaining a cadence of 70 - 80 rpm, with an initial minimal workload for 1 minute. While the participants continued to exercise in the bore of the MRI scanner, the workload was increased by 25 W every minute until the target HR was reached or leg exhaustion occurred. The free-breathing RT imaging began immediately following the cessation of exercise in order to minimize movement artifacts and electrocardiogram mis-triggering.

### Image Analysis

**Figure 3:**
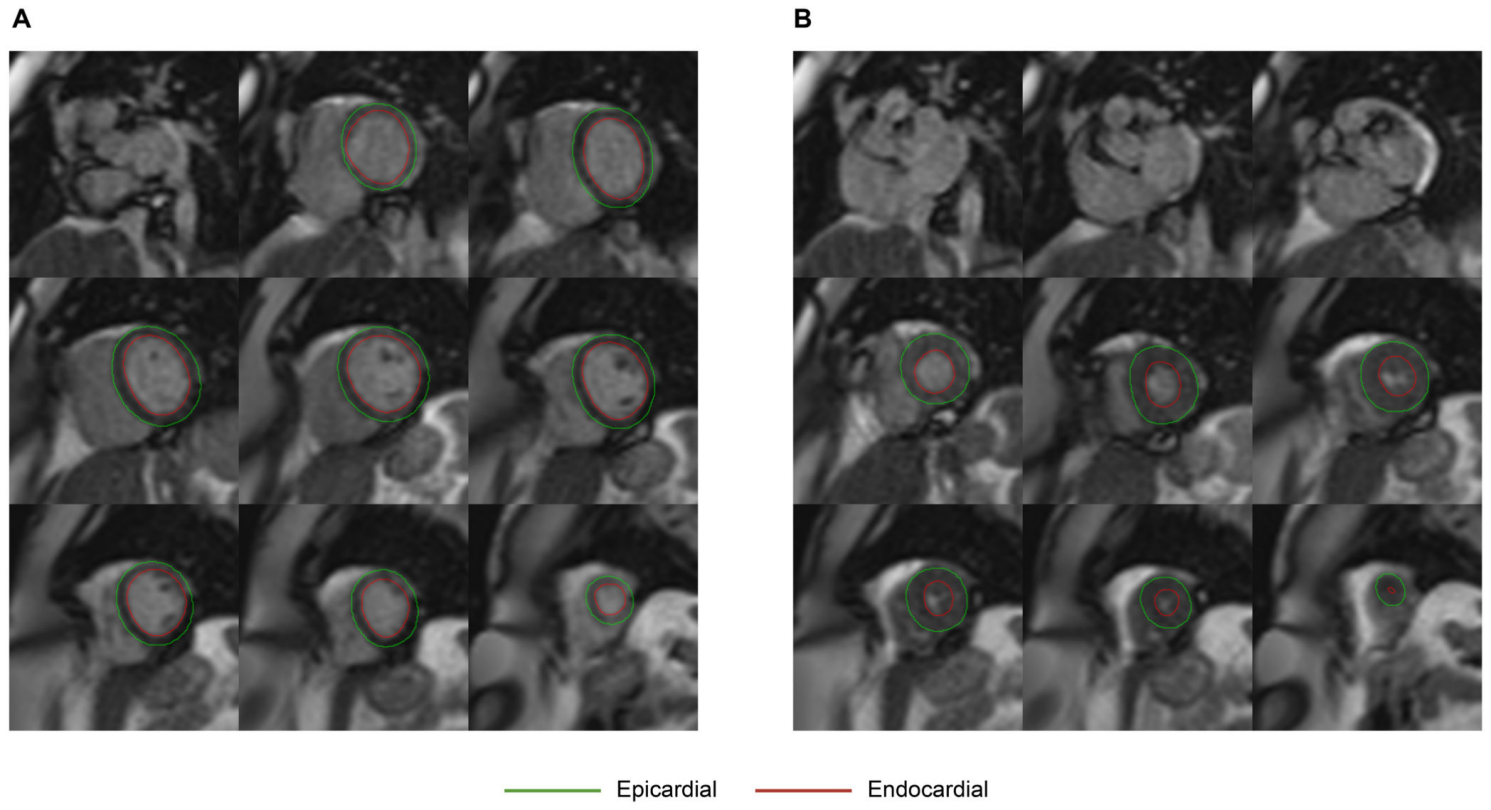
Real-time cine imaging. Example of exercise real-time short-axis cine images without contrast from a 52-year-old man at a) end diastole and b) end systole. Epicardial contours are shown in green and the endocardial contours in red.

In this study, only breath-held cine images and free-breathing RT images were analyzed. All images were analyzed using cvi42 (Circle Cardiovascular Imaging Inc., Calgary, Canada, version 5.16). End-systolic and end-diastolic frames were manually selected. Subsequently, left ventricular endo- and epicardial borders were traced using automated deep learning segmentation within cvi42 and were then visually inspected and corrected if necessary (Figure 3, Video S2 and S3).^12^ The automated segmentation generally performed well with only minor manual adjustments, primarily in the apical and basal regions, as needed. Papillary muscles were included in the left ventricular blood pool. Absolute values were indexed to body surface area using the DuBois method. Cardiac output and cardiac index were calculated using the maximal heart rate achieved. Wall strain was not assessable on the RT data. Readers were a cardiology fellow (RS; 2 years of experience) and a senior cardiology fellow (MS; 7 years of experience).

### Intra- and Inter-observer Reliability

Inter- and intra-observer variability were evaluated to assess reliability of the cardiac MRI measurements. Fifty participants were randomly selected and re-analyzed after an interval of 3 months by the two operators blinded to each other’s analysis.

### Statistical Analysis

Statistical analyses were performed using R (version 4.2.1, R Core Team, Vienna, Austria) and Python (version 3.8.5, Python Software Foundation, Delaware, United States). Variables were expressed as percentages, if categorical, and as mean ± standard deviation, if continuous. Bland-Altman plots were used to quantify the agreement between RT and breath-held cines at rest. Intra-class correlation coefficient (ICC) was used to assess intra- and inter-observer reliability and categorized as poor (<0.5), moderate (0.5-0.75), good (0.75-0.9), and excellent (>0.9).^13^ Reference ranges were defined as the 95% prediction interval of the Student’s t-distribution. Ranges were stratified by sex and age groups (20-39, 40-59, 60-79 years). Comparisons were adjusted for age as a continuous variable unless stratified by age group. Mixed linear models were used to compare left ventricular parameters between rest and exercise and between sexes, and one-way analysis of variance (ANOVA) was used to compare parameters across age groups. Post hoc testing used Tukey honest significant difference test. The t-tests were two-sided and the ANOVA one-sided. Adjustments for multiple comparisons were performed using the Benjamini-Hochberg procedure. A *P* value ≤ 0.05 was considered significant.

## Results

### Participant Characteristics

**Figure 4:**
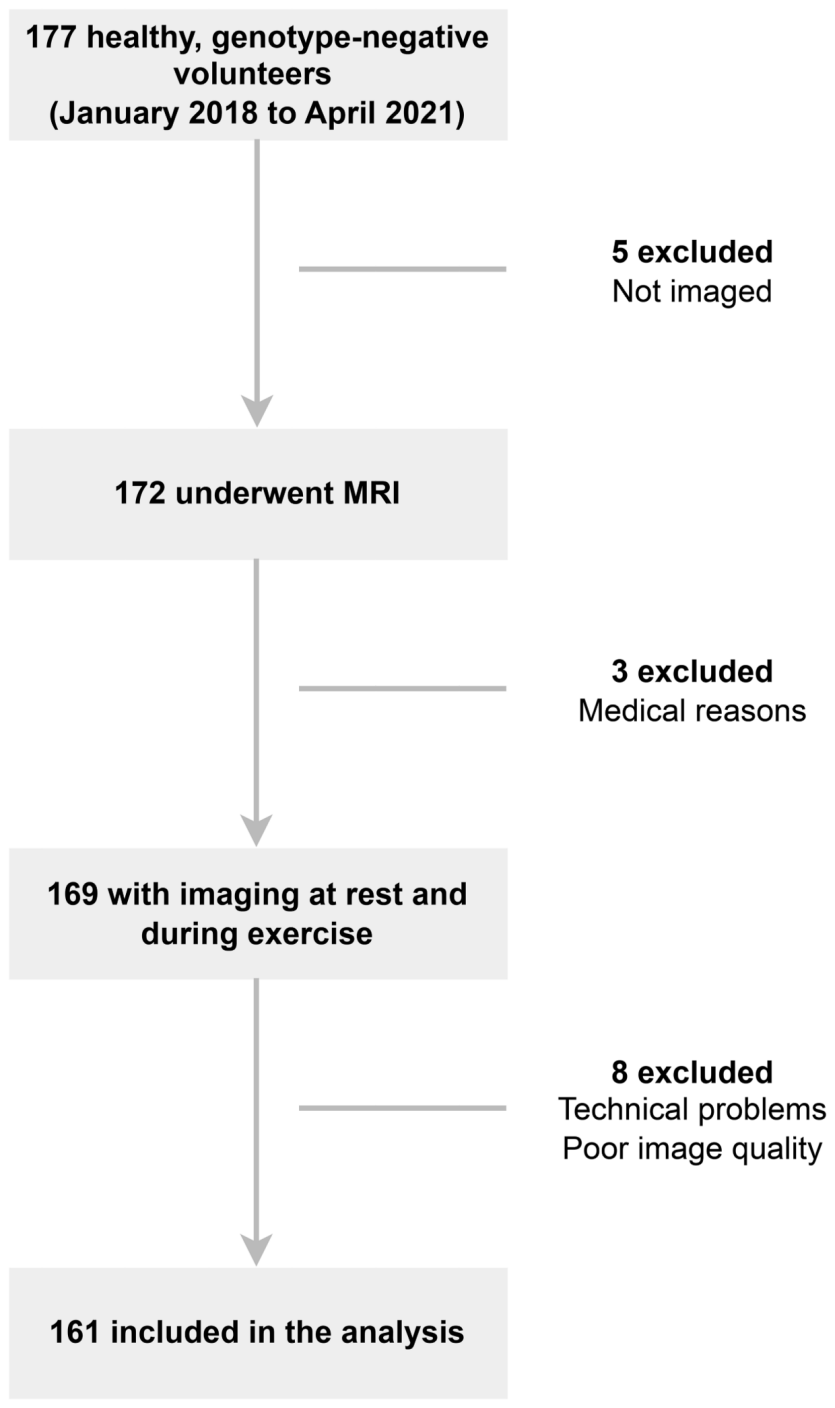
Study flowchart.

A total of 177 healthy, genotype-negative volunteers were enrolled, of whom 161 were included in the sub-study (Figure 4). Five participants did not undergo MRI, three participants were excluded for persistent tachycardia or incidental pleural effusions, and 8 for poor image quality. The mean age was 49±14 years (range 22-77 years), with 70% (114/161) of participants being White and 53% (85/161) female. Baseline characteristics are shown in Table 1. Participants reported the following physical activity levels during enrollment for the original study from which they were drawn: 7% (12/161) engaged in no regular exercise, 40% (65/161) engaged in light activity, 36% (58/161) engaged in moderate physical activity, and 16% (26/161) engaged in more than 5 hours of exercise per week.^14^ The RT images were typically acquired over 27 seconds during which time there was a mean decrease in HR of 12 beats per minute from peak exercise. Examples of the image quality achieved using the free-breathing RT sequence immediately post exercise are shown in Supplementary Video S2 and S3.

### Left Ventricular Reference Values

Reference ranges for absolute and relative change in left ventricular parameters during exercise are stratified by gender and age (male participants: Table 2, female participants: Table 3). The “lower” value represents the lower end of the 95% prediction interval, the “mean” value represents the average change, and the “upper” value represents the upper end of the 95% prediction interval.

### Left Ventricular Response to Exercise

For the cohort as a whole, exercise caused an increase in HR (64±9 bpm vs 133±19 bpm, P < 0.001), stroke volume (SV) (82±18 ml vs 102±25 ml, P < 0.001), ejection fraction (EF) (59±6% vs 69±7%, P < 0.001), and cardiac output (5.2±1.1 l/min vs 13.5±3.9 l/min, P < 0.001). The increase in EF and SV was predominantly mediated by a reduction in end-systolic volume (ESV) (58±18 ml vs 46±15 ml, P < 0.001), with a smaller increase in end-diastolic volume (EDV) (140±32 ml vs 148±35 ml, P < 0.001).

### Sex Differences

**Figure 5:**
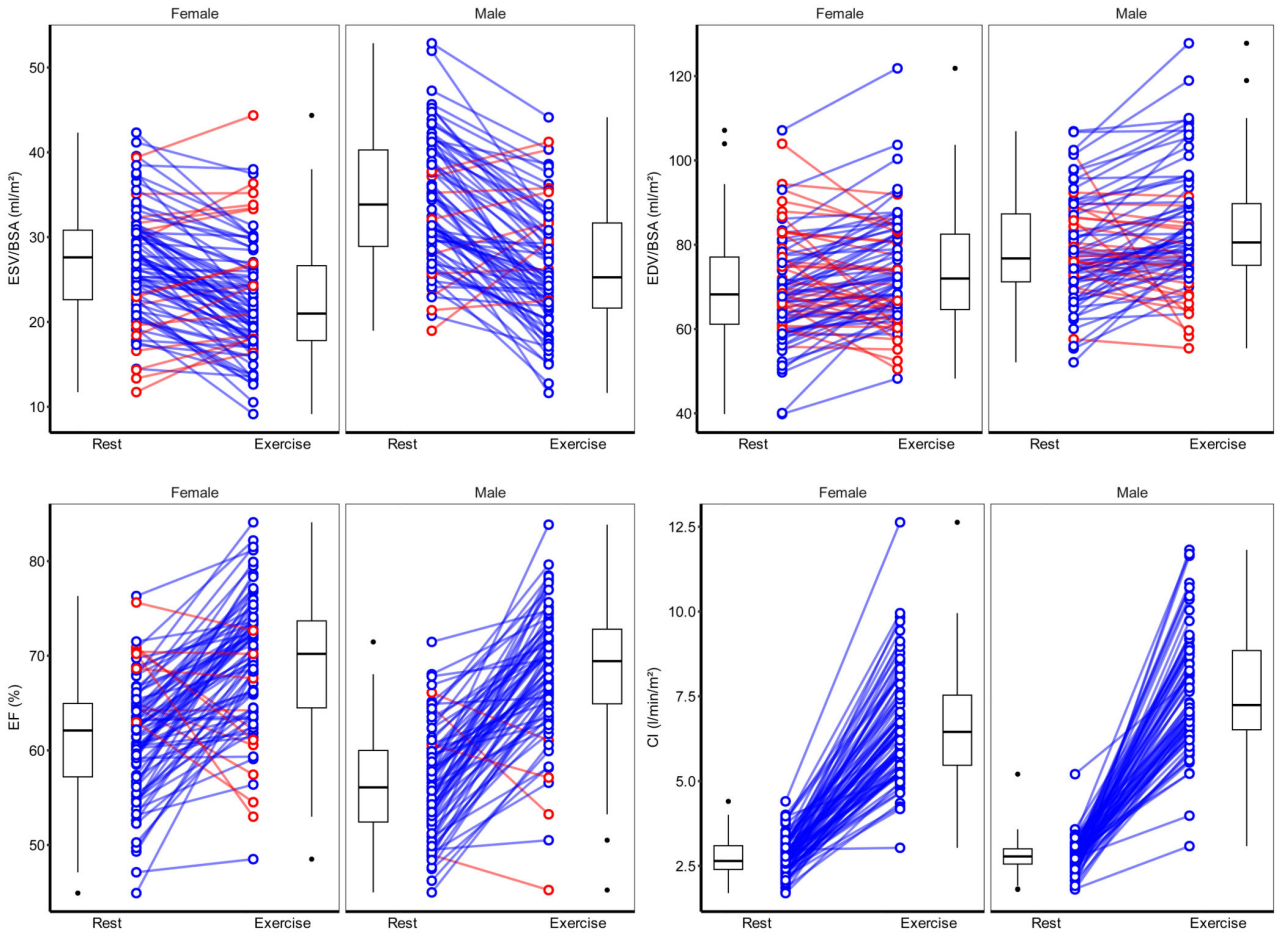
Left ventricular parameters at rest and exercise stratified by sex. Lines join corresponding values. Boxes show median and inter-quartile range (IQR), and whiskers 1.5*IQR. Individual direction of response shown in blue (same) or red (different) with respect to average change. EDV, end-diastolic volume; ESV, end-systolic volume; EF, ejection fraction; CI, cardiac index; BSA, body surface area. n = 161.

Comparisons between left ventricular parameters at rest and exercise are shown in Table 4 and Table S1. Stratified plots of participant-level response to exercise are shown in Figure 5, S1, and S7 to S10. In summary, male participants had higher resting indexed EDV (79±12 ml/m^2^ vs 70±13 ml/m^2^, *P* < 0.001) and ESV (34±7 ml/m2 vs 27±7 ml/m^2^, *P* < 0.001) than female participants, as well as a higher HR increase (72±22 bpm vs 65±18 bpm, *P* < 0.001). Males achieved a higher indexed SV (57±11 ml/m^2^ vs 51±9 ml/m^2^, *P* < 0.001) through greater left ventricular emptying (change in indexed ESV: -8±7 ml/m^2^ vs -4±5 ml/m^2^, *P* = 0.001).

### Age Group Differences

**Figure 6:**
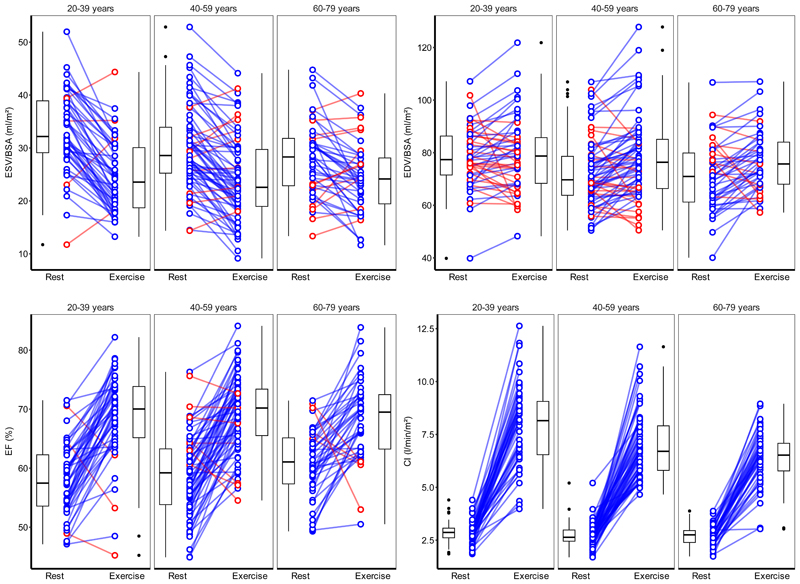
Left ventricular parameters at rest and exercise stratified by age group. Lines join corresponding values. Boxes show median and inter-quartile range (IQR), and whiskers 1.5*IQR. Individual direction of response shown in blue (same) or red (different) with respect to average change. EDV, end-diastolic volume; ESV, end-systolic volume; EF, ejection fraction; CI, cardiac index; BSA, body surface area. n = 161.

Comparisons between left ventricular parameters at rest and exercise stratified by age group are shown in Tables S3 to S6, Figure 6, and Figure S1 to S6. In summary, HR response to exercise declined with age in both sex groups (male and female: *P* < 0.01). Older female participants showed less ventricular emptying (change in indexed ESV, male: *P* = 0.07; female: *P* = 0.012) but male participants achieved an increase in indexed EDV (male: *P* = 0.035; female: *P* = 0.6). There was no evidence of a difference in exercise EF between sexes (*P =* 0.08), and cardiac index values tended to increase across age groups (male: *P* = 0.07; female: *P* = 0.006).

### Variability in stroke volume response

In this study, 142 individuals had an increase in their left ventricular SV during exercise while 19 did not. Age and sex distributions were similar across both groups (*P* = 0.86 for age groups; *P* = 0.47 for gender). Significant differences were observed in several LV parameters when comparing the two groups. Normalized EDV at rest was lower in those with a SV response (73±13 ml/m^2^ vs 82±12 ml/m^2^, *P =* 0.004), accompanied by an increase in EDV from rest to exercise (11±12 ml/m^2^ vs -6±8 ml/m^2^, *P* < 0.001), and a less pronounced HR response (107±42% vs 128±34%, *P <* 0.001). In contrast, the change in normalized ESV from rest to exercise did not differ between groups (-6±6 ml vs -4±9 ml, *P* = 0.23). There was no consistent effect of activity level on change in SV.

### Data Reliability

Bland-Altman plots (Figure S11 to S19) comparing resting RT imaging with breath-held cines showed a good level of agreement and no measurement bias. Intra- and inter-observer reliability testing demonstrated good to excellent agreement for all measured variables (Table S7).

## Discussion

This study reports the effects of exercise in healthy volunteers assessed with exercise cardiac MRI and provides corresponding reference ranges stratified by age and sex. In our cohort of 161 adults without known cardiovascular disease (mean age, 49±14 years; 85 female), exercise caused an increase in left ventricular cardiac output (5.2±1.1 l/min vs 13.5±3.9 l/min, P < 0.001), SV (82±18 ml vs 102±25 ml, *P* < 0.001), and EF (59±6% vs 69±7%, P < 0.001). These changes were driven by a increase in HR (64±9 bpm vs 133±19 bpm, *P* < 0.001), an increase in left ventricular EDV (140±32 ml vs 148±35ml, *P* < 0.001), and a decrease in left ventricular ESV (58±18 ml vs 46±15 ml, P < 0.001). Defining normal ranges for left ventricular parameters will enable exercise cardiac MRI findings to be placed in the context of normal variation and will facilitate non-pharmacological, quantitative assessment of functional reserve. The availability of this normative data will assist with the clinical interpretation of exercise cardiac MRI as it becomes more widely used in both research settings and clinical practice. The study also highlights sources of individual variation in response to supine exercise, which should be taken into account when interpreting the clinical findings.

Cardiac MRI is considered the reference standard for assessing resting cardiac function and a single exam provides quantitative information about cardiac structure, tissue characteristics and blood flow.^15^ Cardiac MRI has multiple Class I and Class 2a recommendations for pharmacological functional testing,^16^ and more recently, exercise cardiac MRI has emerged as a non-pharmacological assessment of physiological reserve that is safe and cost effective.^1,17^ The EXACT trial showed the potential of using treadmill exercise cardiac MRI in patients at risk for coronary artery disease, demonstrating a strong agreement with coronary angiography and outperforming SPECT.^18^ Additionally, the EMPIRE study showed excellent agreement between supine exercise cardiac MRI and invasive coronary fractional flow reserve.^19^ The use of exercise cardiac MRI also provides a non-invasive approach to identify heart failure with preserved EF in cases where both clinical and echocardiographic findings are inconclusive.^20^ Further applications of exercise cardiac MRI include differentiating between athlete’s heart and dilated cardiomyopathy,^21,22^ as well as stratification of patients with pulmonary hypertension.^23^ While these trials show that exercise cardiac MRI has diagnostic value in patients with cardiometabolic comorbidities, underlying causes of relative exercise intolerance may cause variation in the ability to reach target HR.

The widening availability and indications for exercise cardiac MRI highlight the need for published normal ranges, which have not previously been reported for this technique.^5^ There are variations in practice regarding exercise regimens, imaging protocols and post-processing as this is still an emerging technique. For the study sample, we recruited healthy volunteers in the UK that had been pre-screened for cardiovascular disease, including incidental cardiomyopathy-associated rare variants. Study participants had an age range from 21 to 77 years; there were similar numbers of male and female participants and 70% of total participants were White. Analyses were stratified by age and sex. We did not have a sufficient sample size to stratify by race and ethnicity. The ranges therefore represent a community-based urban reference population free of known disease. The exercise regimen is based on reaching an age-predicted maximal HR to individualize exercise intensity,^24^ although some studies have used upright cardiopulmonary exercise testing to adjust target power output,^23^ or used a single target HR.^2^ Our protocol has the practical advantage of being personalized while not requiring pre-test cardiopulmonary exercise testing. There are also a variety of exercise equipment and protocols available including treadmill exercise outside the magnet. While MRI-conditional cycle ergometers are expensive and currently less widely available, they offer a means of accurately controlling exercise intensity while inside the bore of the magnet with immediate imaging reducing potential sources of variability. Of those that underwent exercise cardiac MRI, only 5% were excluded from subsequent analysis. Imaging protocols for exercise cardiac MRI favor RT imaging as the patient is free-breathing after exercise. While accelerated RT sequences continue to be developed to improve temporal resolution and mitigate motion artifact,^25^ we used a product sequence that is widely available, shows strong agreement with gated cine sequences and achieves good reproducibility. To facilitate analysis of end-diastolic and end-systolic frames on RT imaging, we used a reconstruction that produces a consistent number of frames, but this can also be readily achieved without interpolation. For post-processing we used widely available commercial software for automated assessment initially followed by manual refinement. The overall protocol is broadly generalizable and reflects current practice.

A consistent response to exercise has been shown in meta-analyses of exercise cardiac MRI across different field strengths, exercise protocols, and imaging techniques.^6^ Our cohort demonstrated a robust response to supine exercise, with almost a 250% increase in cardiac output, achieving at least the maximal response to similar protocols.^21^ While only 38% of participants achieved their target HR on exercise, this is consistent with prior studies comparing stress protocols where no participants were able to achieve criteria for VO2max while exercising supine whilst all did when upright.^26,27^ Such hemodynamic differences between supine and upright exercise suggest that a protocol specific HR goal may be appropriate. Overall, EDV showed only a modest and variable increase on exercise, while there was a significant rise in HR and decrease in ESV. Older adults, with a reduced HR response and less systolic reserve, relied on greater ventricular filling to maintain exercise cardiac output. Higher HR in younger adults during exercise, with decreased filling time, could account for the limited increase in EDV during physical activity.^28^ In the oldest age groups, a decline in diastolic relaxation could also contribute to a blunted rise in cardiac output.^29^ Male participants had higher indexed resting and exercise volumes than female participants and a greater absolute and relative response to exercise, findings consistent with other studies.^30^ The rise in HR was similar between sexes, and a higher exercise SV in males was achieved through greater left ventricular emptying. While 88% of participants showed a combined increase in SV and HR, the minority relied on an increase in HR alone during exercise. Previous exercise studies have shown that changes in cardiac index are predominantly driven by HR,^6,31^although we show a variable contribution by SV that is age and sex-dependent. With respect to rising VO_2_, a progressive increase, a plateau with a secondary increase, a flat plateau, and a plateau with a drop in SV have all been reported.^32^ Some of this variability in healthy adults may be accounted for by aerobic fitness levels, reflecting the relationship between parameters of fitness (VO_2_max) and myocardial contractility (EF or SV) with the pattern of exercise-dependence of SV. We also demonstrated that it was feasible to incorporate exercise cardiac MRI into a full clinical protocol, including tissue characterization and late enhancement, which could be completed within 60 minutes with a low technical failure rate. Although the study included only healthy volunteers, this finding suggests exercise cardiac MRI could be incorporated into routine cardiac MRI practice. Suitable quality control measures would be recommended that may include validating accelerated cine sequences, ensuring that artifacts do not affect volumetry across exercise intensities, and ensuring that segmentation approaches are accurate and manually corrected if necessary.

Our study had limitations. First, our cohort comprised mainly white participants, which could limit the generalizability of our reference ranges to other races and ethnicities. Second, baseline fitness and lifestyle can significantly affect cardiac function even in non-athletes, and our cohort represented a healthy urban population that included those performing regular exercise.^14^ Third, the study was conducted at a single center and we did not assess the variability due to other scanners or protocols. We used widely available sequences and analysis, but novel sequences to increase temporal and spatial resolution could potentially achieve better reliability and functional characterisation.^33^ Images were acquired immediately after peak exercise during which time there was modest decrease in heart rate. Acquisition times were comparable with published studies for in-scanner exercise cardiac MRI and also obviated delays in transfer compared to treadmill protocols.^1^ Fourth, we did not assess test-retest performance due to the effects of fatigue and were not able to recall participants for a repeat visit. Fifth, we used self-reported measures of physical activity that have acceptable validity when compared to VO_2_ max.^34^ A normal values study in almost 500 health young adults using the same analysis software had comparable resting values (e.g., male/female indexed LVEDV 89/78 vs 79/70 ml/m^2^, LVESV 32/27 vs 35/28 ml/m^2^).^35^ Finally, the higher end-diastolic values in our analysis may be due to including papillary muscles in the cavity volume and the method of volumetry may influence the values obtained.

In conclusion, this study establishes the normal response to exercise assessed by exercise cardiac MRI and provides left ventricular adult reference ranges in a community-based cohort. Quantitative evaluation of exercise cardiac MRI using these normative data will enable better differentiation between health and disease in clinical practice.

## Supplementary Material

Supplementary Material

## Figures and Tables

**Table 1:** Participant characteristics.

	Male			Female		
	20 - 39 years n = 26	40 - 59 years n = 32	60 - 79 years n = 18	20 - 39 years n = 24	40 - 59 years n = 35	60 - 79 years n = 26
Age (years)	32 ± 5	49 ± 6	66 ± 5	31 ± 4	52 ± 5	67 ± 4
White	12 (46)	23 (72)	16 (87)	16 (67)	26 (74)	21 (81)
Weight (kg)	81 ± 13	86 ± 16	85 ± 14	68 ± 12	71 ± 14	63 ± 12
Height (cm)	179 ± 6	179 ± 7	178 ± 5	166 ± 6	163 ± 6	162 ± 6
BSA (m^2^)	2.0 ± 0.18	2.05 ± 0.19	2.03 ± 0.17	1.76 ± 0.16	1.77 ± 0.16	1.67 ± 0.14
BMI (kg/m^2^)	25.3 ± 3.6	26.8 ± 4.2	26.7 ± 4.0	24.7 ± 4.2	26.8 ± 5.9	24.3 ± 4.9
Systolic Blood Pressure (mmHg)	127 ± 12	132 ± 13	138 ± 17	115 ± 9	123 ± 13	135 ± 22
Diastolic Blood Pressure (mmHg)	77 ± 8	83 ± 8	83 ± 11	72 ± 7	79 ± 9	78 ± 10
Heart Rate Baseline (beats per minute)	62 ± 7	65 ± 13	61 ± 9	66 ± 7	64 ± 7	64 ± 6
Heart Rate Max (beats per minute)	150 ± 16	130 ± 20	123 ± 16	139 ± 16	130 ± 12	121 ± 14
Target HR reached?	13 (50)	10 (31)	10 (56)	6 (25)	11 (31)	11 (42)
Percentage of target HR reached	94 ± 10	90 ± 13	95 ± 12	88 ± 11	91 ± 9	94 ± 11
Percent of Predicted Max HR reached	80 ± 8	76 ± 11	81 ± 10	74 ± 9	77 ± 8	80 ± 10
Increase in HR	141 ± 25	118 ± 61	110 ± 37	109 ± 34	103 ± 31	84 ± 28
Decrease in HR during scanning	11 ± 5	11 ± 7	14 ± 7	11 ± 5	12 ± 8	9 ± 7
Wattage Max (W)	159 ± 31	139 ± 39	126 ± 32	120 ± 26	98 ± 26	83 ± 27
Activity Level						
Sedentary	2 (8)	1 (3)	0 (0)	1 (4)	6 (17)	2 (8)
Lightly Active	7 (27)	13 (41)	8 (44)	9 (38)	15 (43)	13 (50)
Moderately Active	14 (54)	11 (34)	8 (44)	5 (21)	13 (37)	7 (27)
Very Active	3 (12)	7 (22)	2 (11)	9 (38)	1 (3)	4 (15)

Note.— Continuous variables are expressed as mean ± standard deviation and categorical variables as number of participants (percentage) — Demographic and physiological parameters for the study participants. Heart rate shown at rest and peak exercise. BSA, body surface area; BMI, body mass index; bpm, beats per minute; HR, heart rate. Activity levels defined as: Sedentary (almost entirely sedentary with no regular exercise), Lightly active: light physical activity or exercise for 1 to 3 hours per week, Moderately active: physical activity or exercise between 3 and 5 hours per week, Very Active: engaged in more than 5 hours of exercise per week.

**Table 2:** Absolute and relative reference ranges for left ventricular parameters on exercise by age group in male participants.

	20 - 39 years			40 - 59 years			60 - 79 years		
	lower	mean	upper	lower	mean	upper	lower	mean	upper
EDV - ml	-10 (-5%)	0 (0%)	9 (6%)	11 (6%)	18 (11%)	24 (16%)	0 (0%)	11 (7%)	21 (14%)
EDV/BSA - ml/m2	-5 (-5%)	0 (0%)	5 (6%)	5 (6%)	9 (11%)	12 (16%)	0 (0%)	5 (7%)	10 (14%)
ESV - ml	-26 (-34%)	-21 (-27%)	-15 (-19%)	-20 (-27%)	-15 (-20%)	-11 (-14%)	-18 (-28%)	-11 (-15%)	-3 (-2%)
ESV/BSA - ml/m2	-13 (-34%)	-10 (-27%)	-8 (-19%)	-9 (-27%)	-7 (-20%)	-5 (-14%)	-9 (-28%)	-5 (-15%)	-2 (-2%)
SV - ml	11 (13%)	20 (23%)	29 (34%)	26 (30%)	33 (39%)	40 (47%)	14 (15%)	22 (24%)	29 (33%)
SV/BSA - ml/m2	6 (13%)	10 (23%)	15 (34%)	13 (30%)	16 (39%)	19 (47%)	7 (15%)	10 (24%)	14 (33%)
EF - %	9 (16%)	12 (22%)	15 (28%)	11 (19%)	13 (24%)	15 (29%)	6 (9%)	9 (16%)	13 (22%)
CO - l/min	9.4 (166%)	10.9 (203%)	12.4 (240%)	8.6 (153%)	10.0 (185%)	11.5 (217%)	7.5 (129%)	8.5 (150%)	9.5 (170%)
CI - l/min/m2	4.7 (166%)	5.5 (203%)	6.2 (240%)	4.1 (153%)	4.8 (185%)	5.5 (217%)	3.6 (129%)	4.2 (150%)	4.8 (170%)

Note.— Values represent absolute changes and corresponding percentage changes from rest to exercise — EDV, end-diastolic volume; ESV, end-systolic volume; SV, stroke volume; EF, ejection fraction; CO, cardiac output; CI, cardiac index; BSA, body surface area.

**Table 3:** Absolute and relative reference ranges for left ventricular parameters on exercise by age group in female participants.

	20 - 39 years			40 - 59 years			60 - 79 years		
	lower	mean	upper	lower	mean	upper	lower	mean	upper
EDV - ml	-1 (0%)	4 (3%)	11 (8%)	-1 (0%)	4 (5%)	11 (10%)	2 (3%)	9 (10%)	16 (18%)
EDV/BSA - ml/m^2^	0 (0%)	2 (3%)	6 (8%)	0 (0%)	2 (5%)	6 (10%)	1 (3%)	5 (10%)	9 (18%)
ESV - ml	-16 (-31 %)	-12 (-21%)	-8 (-12%)	-10 (-23%)	-7 (-16%)	-4 (-9%)	-7 (-15%)	-4 (-7%)	0 (0%)
ESV/BSA - ml/m^2^	-9 (-31%)	-7 (-21%)	-4 (-12%)	-5 (-23%)	-4 (-16%)	-2 (-9%)	-4 (-15%)	-2 (-7%)	0 (0%)
SV - ml	10 (14%)	17 (21%)	24 (29%)	5 (11 %)	12 (20%)	19 (30%)	6 (12%)	13 (24%)	21 (36%)
SV/BSA - ml/m^2^	6 (14%)	9 (21%)	13 (29%)	3 (11 %)	7 (20%)	10 (30%)	3 (12%)	8 (24%)	12 (36%)
EF - %	7 (12%)	10 (17%)	13 (22%)	5 (9%)	8 (13%)	10 (18%)	2 (5%)	6 (11%)	9 (16%)
CO - l/min	7.0 (136%)	8.3 (161%)	9.7 (185%)	5.9 (125%)	6.6 (144%)	7.4 (162%)	4.9 (110%)	5.7 (133%)	6.5 (155%)
CI - l/min/m^2^	4.0 (136%)	4.7 (161%)	5.4 (185%)	3.3 (125%)	3.7 (144%)	4.2 (162%)	2.9 (110%)	3.4 (133%)	3.8 (155%)

Note.— Values represent absolute changes and corresponding percentage changes from rest to exercise — EDV, end-diastolic volume; ESV, end-systolic volume; SV, stroke volume; EF, ejection fraction; CO, cardiac output; CI, cardiac index; BSA, body surface area.

**Table 4:** Comparison of rest vs exercise in both sexes.

	Male				Female	
	Rest n = 76	Exercise n = 76	*P* value	Rest n = 85	Exercise n = 85	*P* value
HR (bpm)	64 ± 11	135 ± 21	<0.001	65 ± 7	130 ± 16	<0.001
EDV (ml)	160 ± 27	170 ± 32	<0.001	122 ± 25	129 ± 25	<0.001
EDV/BSA (ml/m2)	79 ± 12	83 ± 14	<0.001	70 ± 13	73 ± 12	<0.001
ESV (ml)	70 ± 16	54 ± 15	<0.001	47 ± 12	39 ± 12	<0.001
ESV/BSA (ml/m2)	34 ± 7	26 ± 7	<0.001	27 ± 7	22 ± 7	<0.001
SV (ml)	90 ± 16	116 ± 24	<0.001	75 ± 16	89 ± 19	<0.001
SV/BSA (ml/m2)	44 ± 8	57 ± 11	<0.001	43 ± 9	51 ± 9	<0.001
EF (%)	56 ± 6	68 ± 7	<0.001	61 ± 6	70 ± 7	<0.001
CO (l/min)	5.7 ± 1.1	15.6 ± 3.7	<0.001	4.8 ± 1.1	11.7 ± 3.0	<0.001
CI (l/min/m2)	2.8 ± 0.5	7.7 ± 1.7	<0.001	2.8 ± 0.5	6.6 ± 1.6	<0.001

Note.— Data presented as mean ± standard deviation — Values adjusted for age. EDV, end-diastolic volume; ESV, end-systolic volume; SV, stroke volume; EF, ejection fraction; CO, cardiac output; CI, cardiac index; BSA, body surface area; bpm, beats per minute.

## Data Availability

Data generated or analyzed during the study are available from the corresponding author by request. The code used to pre-process and analyse the data, as well as aggregated exCMR parameters, is available at (doi: 10.5281/zenodo.10301641).

## Summary Statement

The current study established normal cardiac MRI reference ranges, stratified by sex and age, for left ventricular response to exercise.

## Key Points

1. In healthy adults an increase in cardiac output following exercise was driven by a rise in heart rate (64±9 bpm to 133±19 bpm) increased left ventricular filling (end-diastolic volume: 140±32 ml to 148±35 ml) and greater left ventricular emptying (end-systolic volume: 58±18 ml to 46±15 ml) (all P < 0.001).
2. Sex and age influenced cardiac response to exercise, with male participants achieving greater increase in indexed stroke volume (44±8 ml/m^2^ to 57±11 ml/m^2^ in males vs 43±9 ml/m^2^ to 51±9 ml/m^2^ in females, P = 0.005) and older individuals demonstrating a reduced heart rate response (123±16 bpm in males aged 60-79 vs 150±16 bpm aged 20-39 and 121±14 bpm in females aged 60-79 vs 139±16 bpm aged 20-39, both P < 0.001).
3. Exercise testing was readily integrated into a standard contrast-enhanced cardiac MRI protocol.

## Abbreviations

RT: real-time
EDV: end-diastolic volume
ESV: end-systolic volume
HR: heart rate
SV: stroke volume
EF: ejection fraction
