# Supplementary material for: Establishing Cardiac MRI Reference Ranges Stratified by Sex and Age for Cardiovascular function during Exercise
Source: Radiol Cardiothorac Imaging. Author manuscript; Available in PMC 2025 Aug 1. (PMC12207647; doi:10.1148/ryct.240175)
Supplement: Supplementary Material [file EMS207322-supplement-Supplementary_Material.pdf]

Supplementary Materials

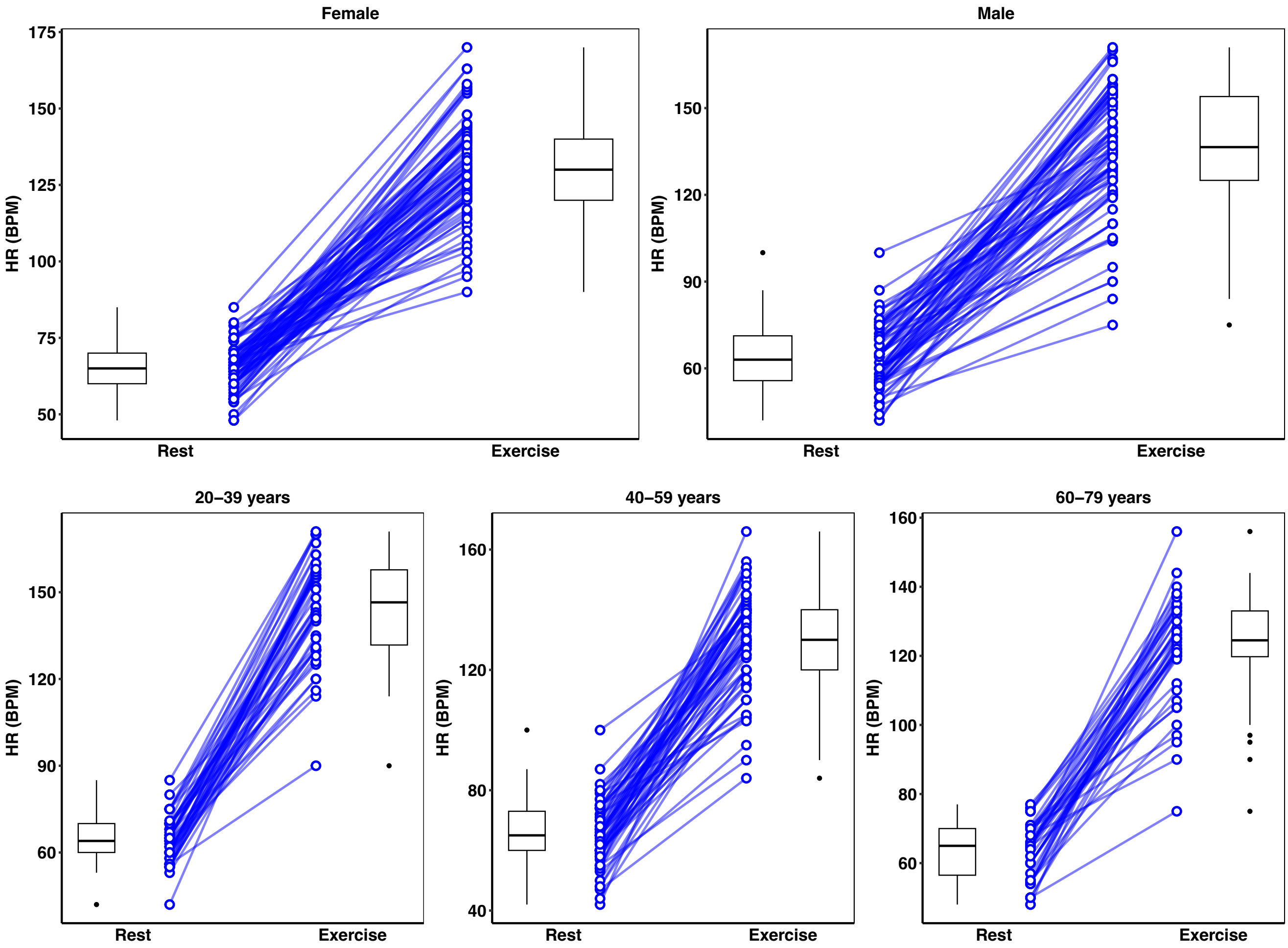

Figure S1. Heart rate at rest and exercise stratified by age and gender. Boxes show median and inter-quartile range (IQR), and whiskers 1.5*IQR. HR, heart rate; BPM, beats per minute. n = 161.

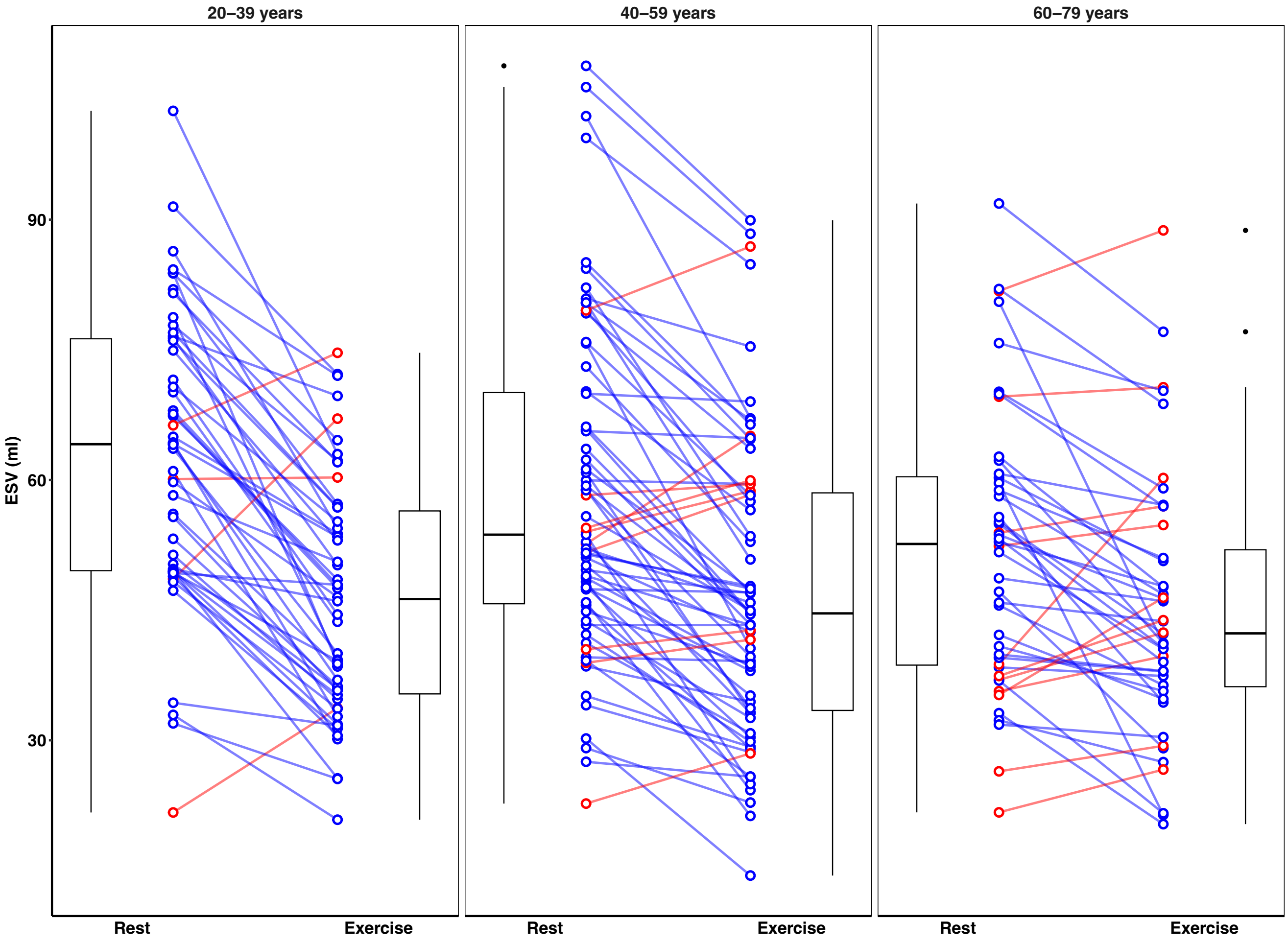

Figure S2. Left ventricular parameters at rest and exercise stratified by age group. Lines join corresponding values. Boxes show median and inter-quartile range (IQR), and whiskers 1.5*IQR. Individual direction of response shown in blue (same) or red (different) with respect to average change. ESV, end-systolic volume. n = 161.

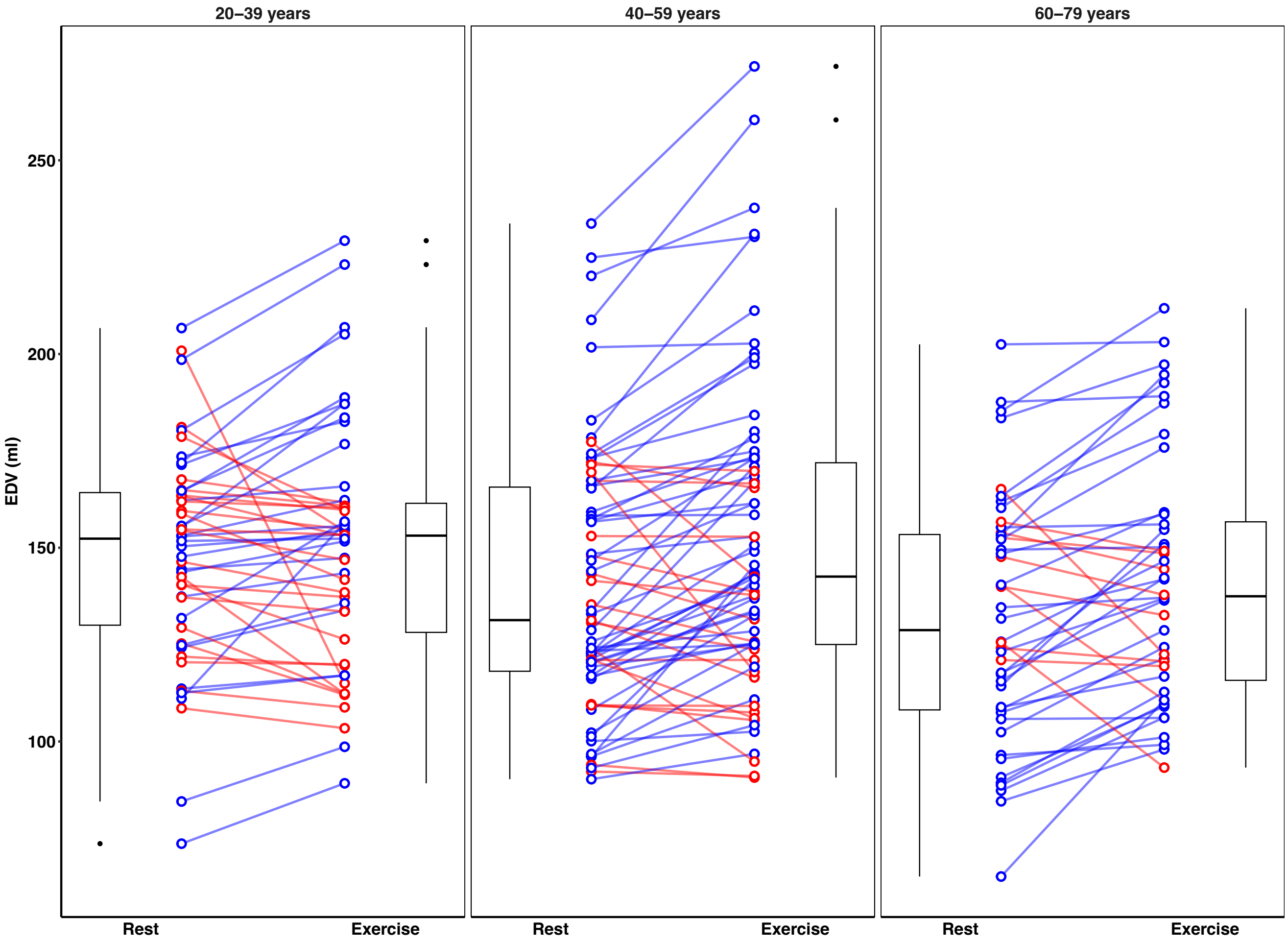

Figure S3. Left ventricular parameters at rest and exercise stratified by age group. Lines join corresponding values. Boxes show median and inter-quartile range (IQR), and whiskers 1.5*IQR. Individual direction of response shown in blue (same) or red (different) with respect to average change. EDV, end-diastolic volume. n = 161.

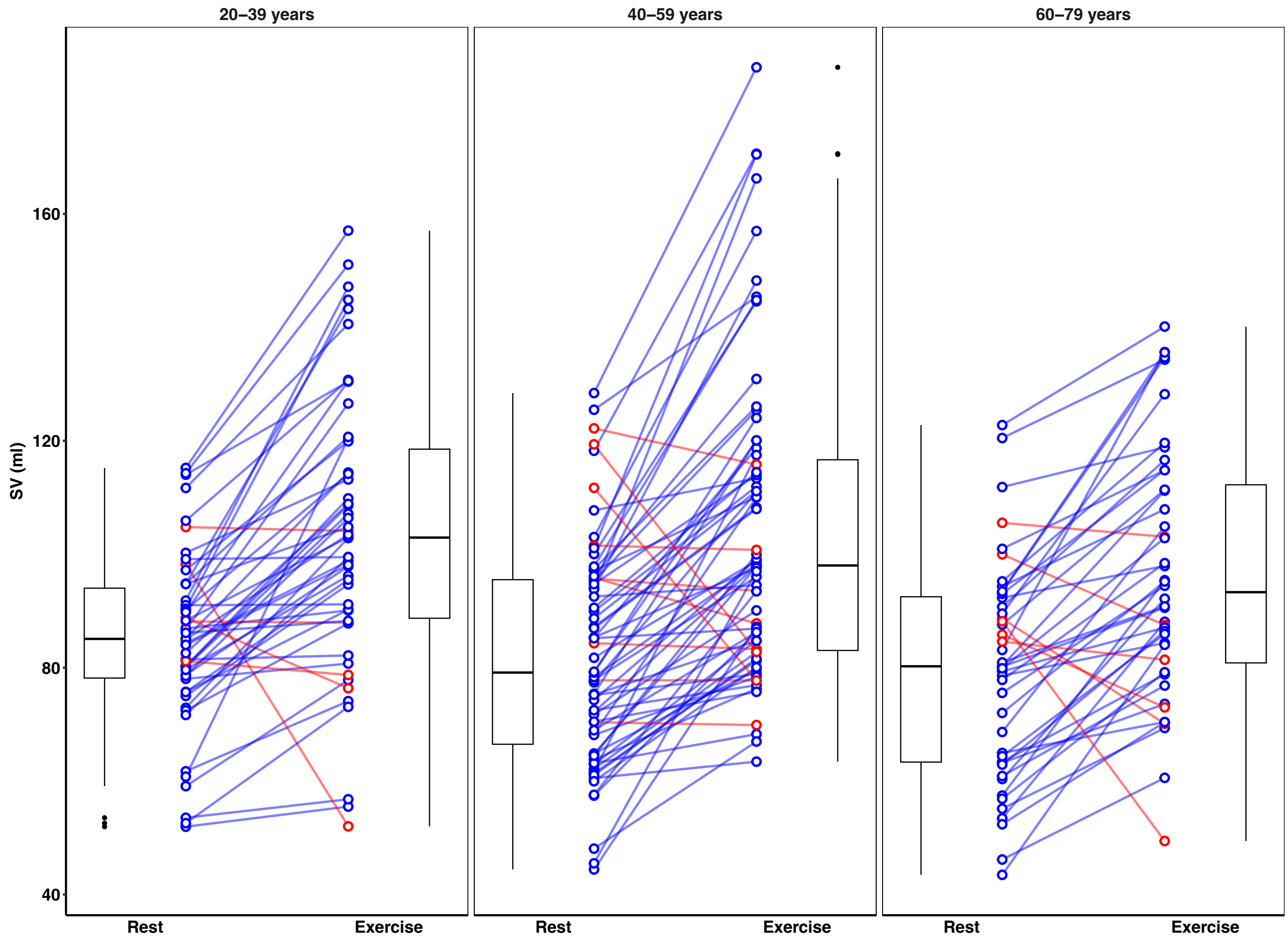

Figure S4. Left ventricular parameters at rest and exercise stratified by age group. Lines join corresponding values. Boxes show median and inter-quartile range (IQR), and whiskers 1.5*IQR. Individual direction of response shown in blue (same) or red (different) with respect to average change. SV, stroke volume. n = 161.

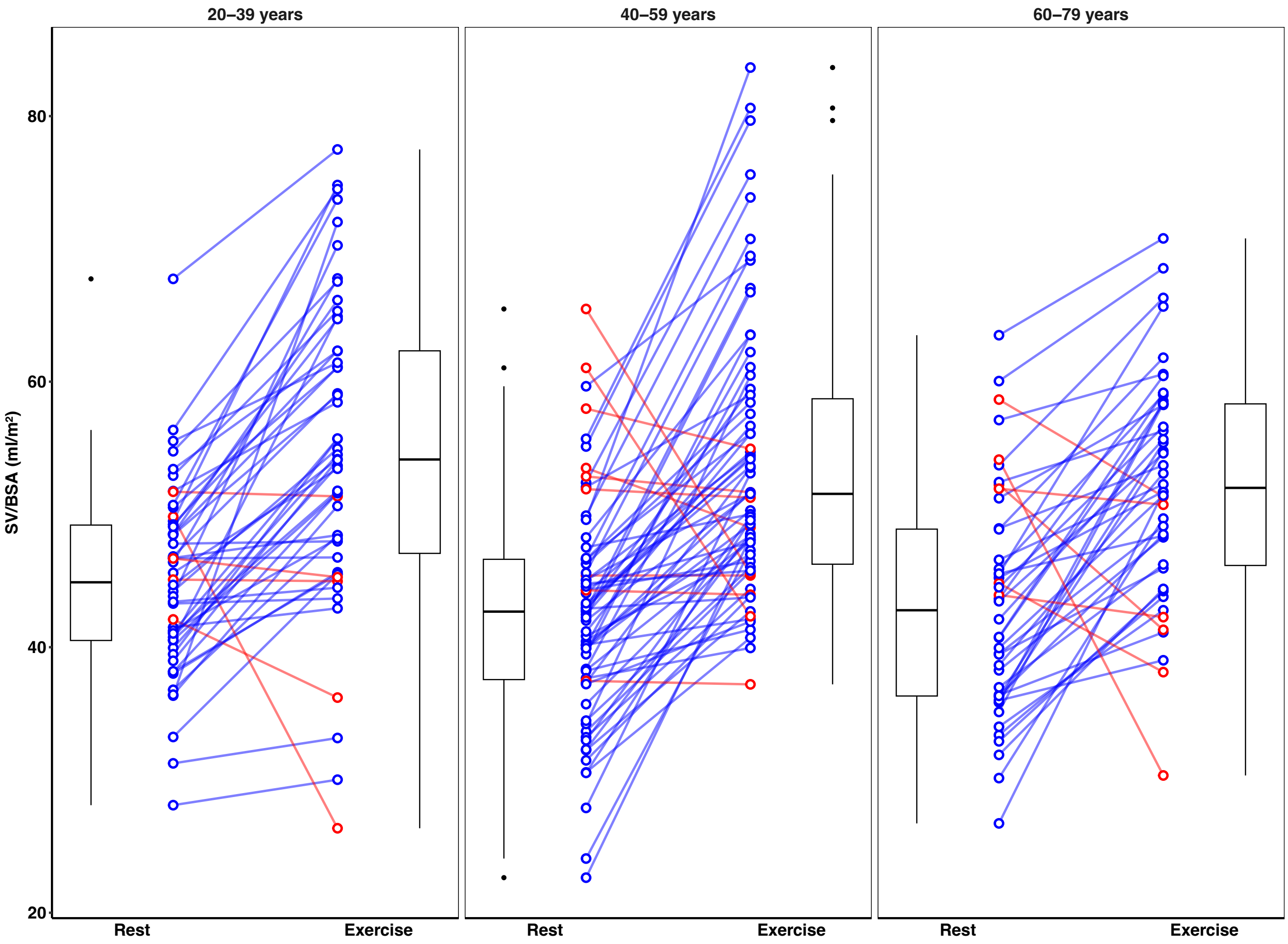

Figure S5. Left ventricular parameters at rest and exercise stratified by age group. Lines join corresponding values. Boxes show median and inter-quartile range (IQR), and whiskers 1.5*IQR. Individual direction of response shown in blue (same) or red (different) with respect to average change. SV, stroke volume; BSA, body surface area. n = 161.

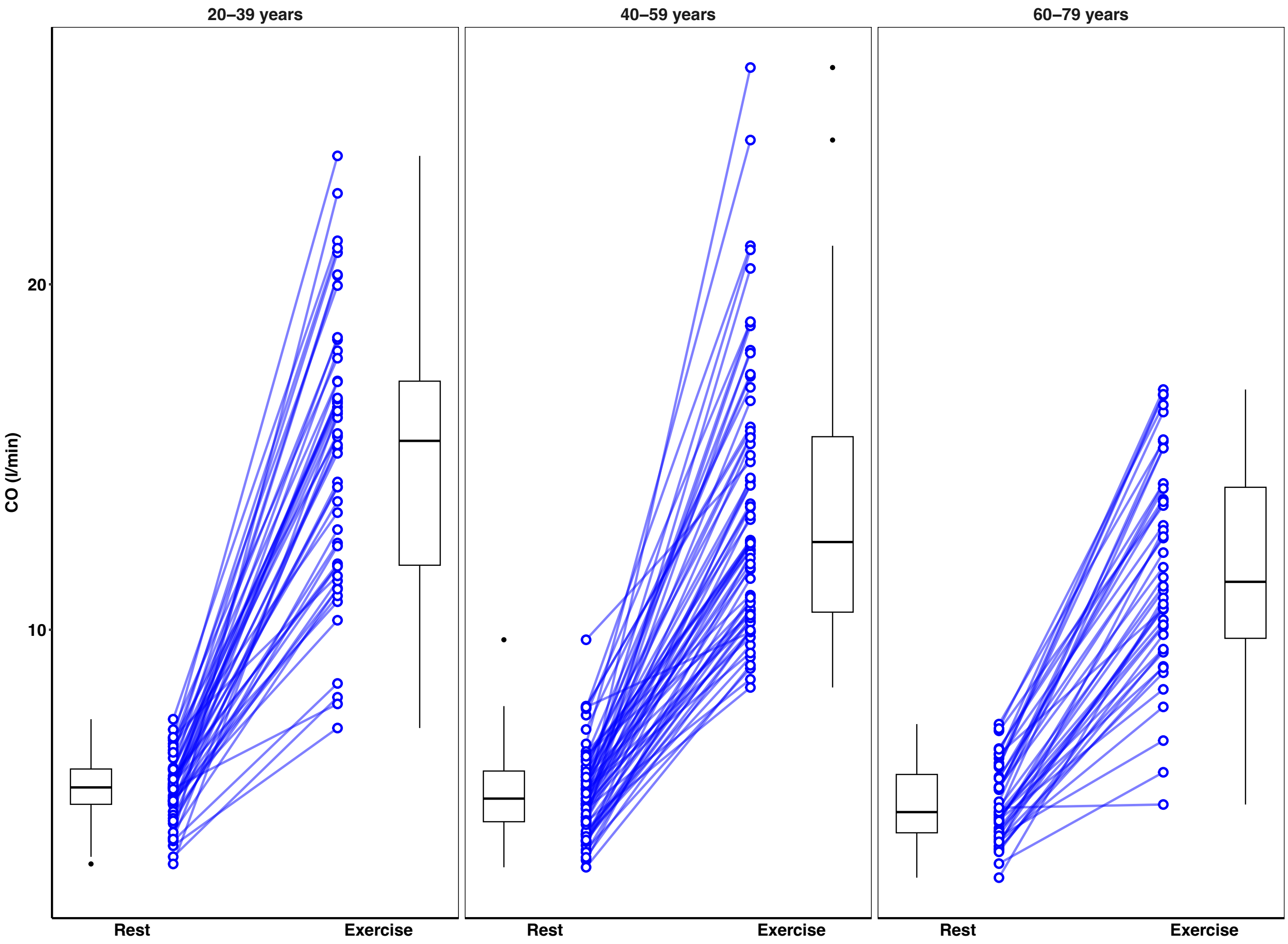

Figure S6. Left ventricular parameters at rest and exercise stratified by age group. Lines join corresponding values. Boxes show median and inter-quartile range (IQR), and whiskers 1.5*IQR. Individual direction of response shown in blue (same) or red (different) with respect to average change. CO, cardiac output. n = 161.

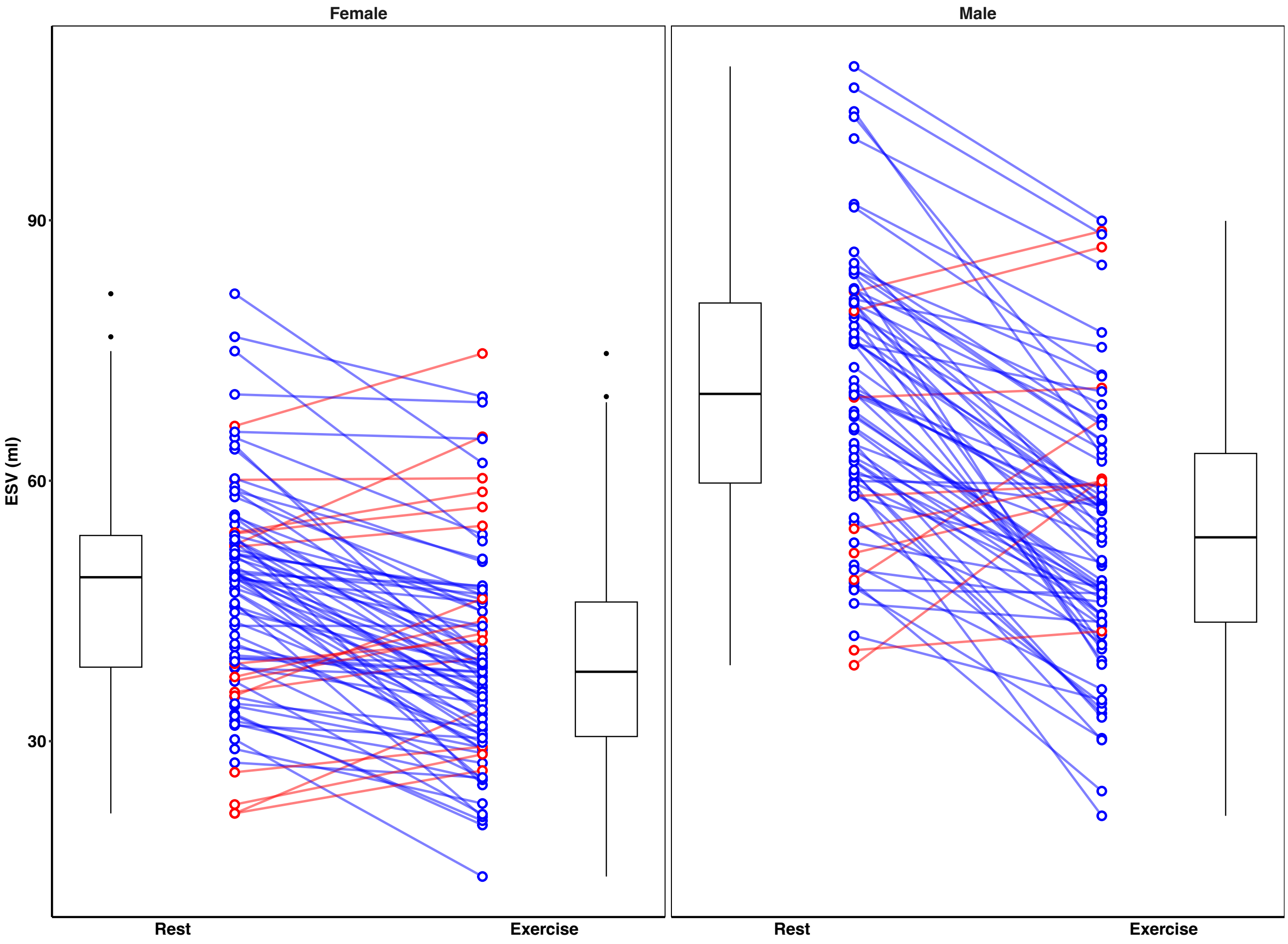

Figure S7. Left ventricular parameters at rest and exercise stratified by gender. Lines join corresponding values. Boxes show median and inter-quartile range (IQR), and whiskers 1.5*IQR. Individual direction of response shown in blue (same) or red (different) with respect to average change. ESV, end-systolic volume. n = 161.

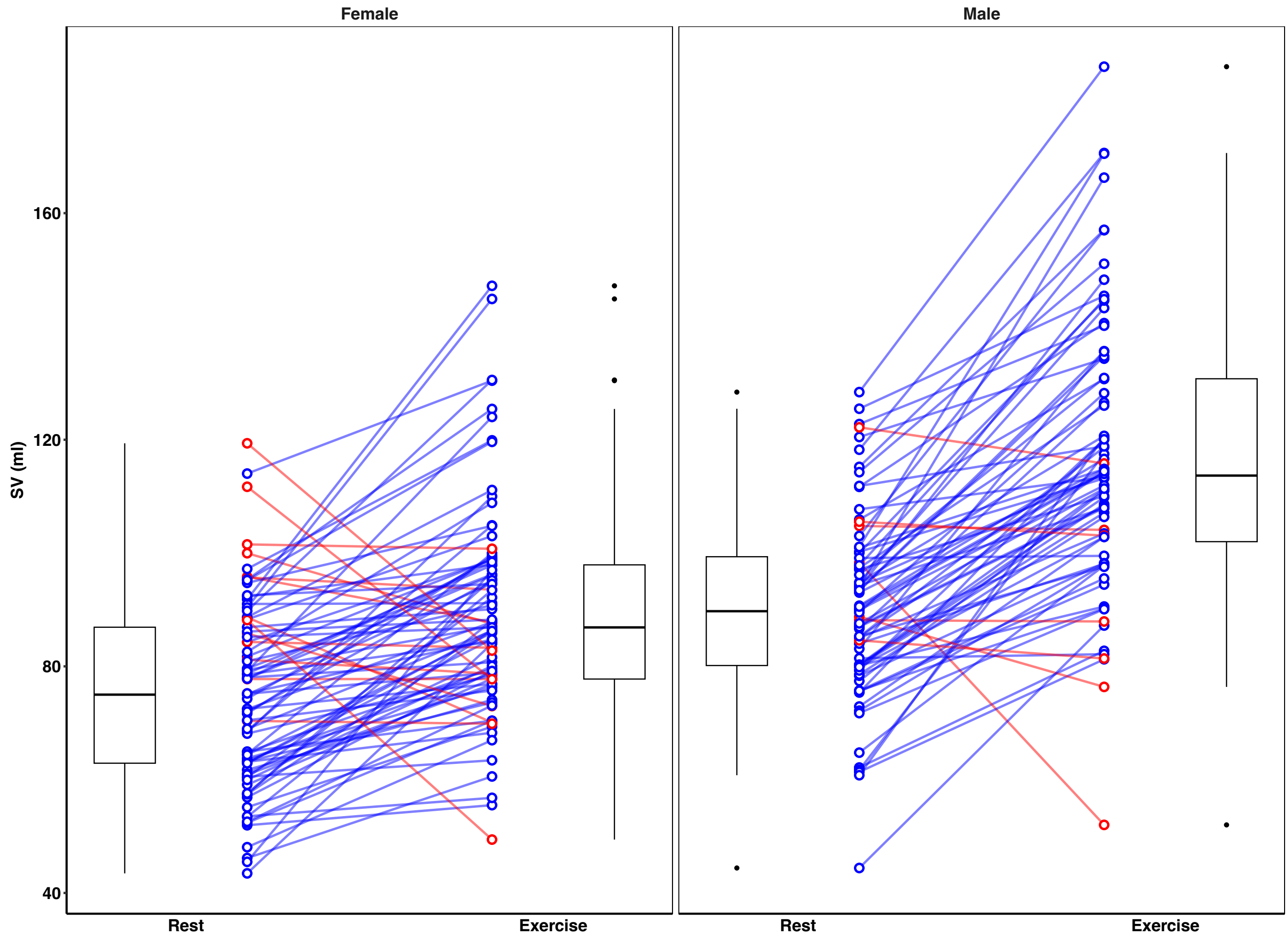

Figure S8. Left ventricular parameters at rest and exercise stratified by gender. Lines join corresponding values. Boxes show median and inter-quartile range (IQR), and whiskers 1.5*IQR. Individual direction of response shown in blue (same) or red (different) with respect to average change. SV, stroke volume. n = 161.

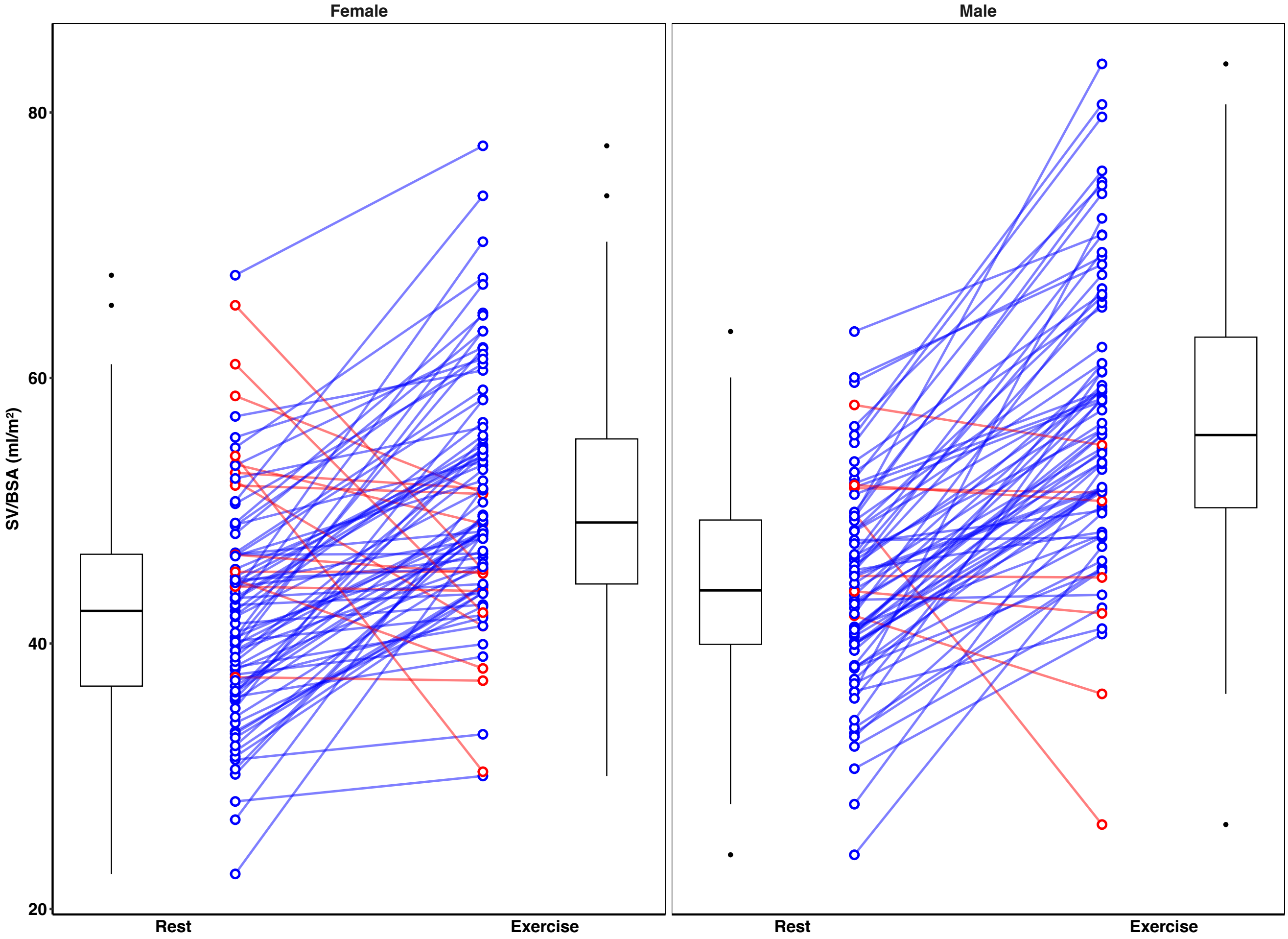

Figure S9. Left ventricular parameters at rest and exercise stratified by gender. Lines join corresponding values. Boxes show median and inter-quartile range (IQR), and whiskers 1.5*IQR. Individual direction of response shown in blue (same) or red (different) with respect to average change. SV, stroke volume; BSA, body surface area. n = 161.

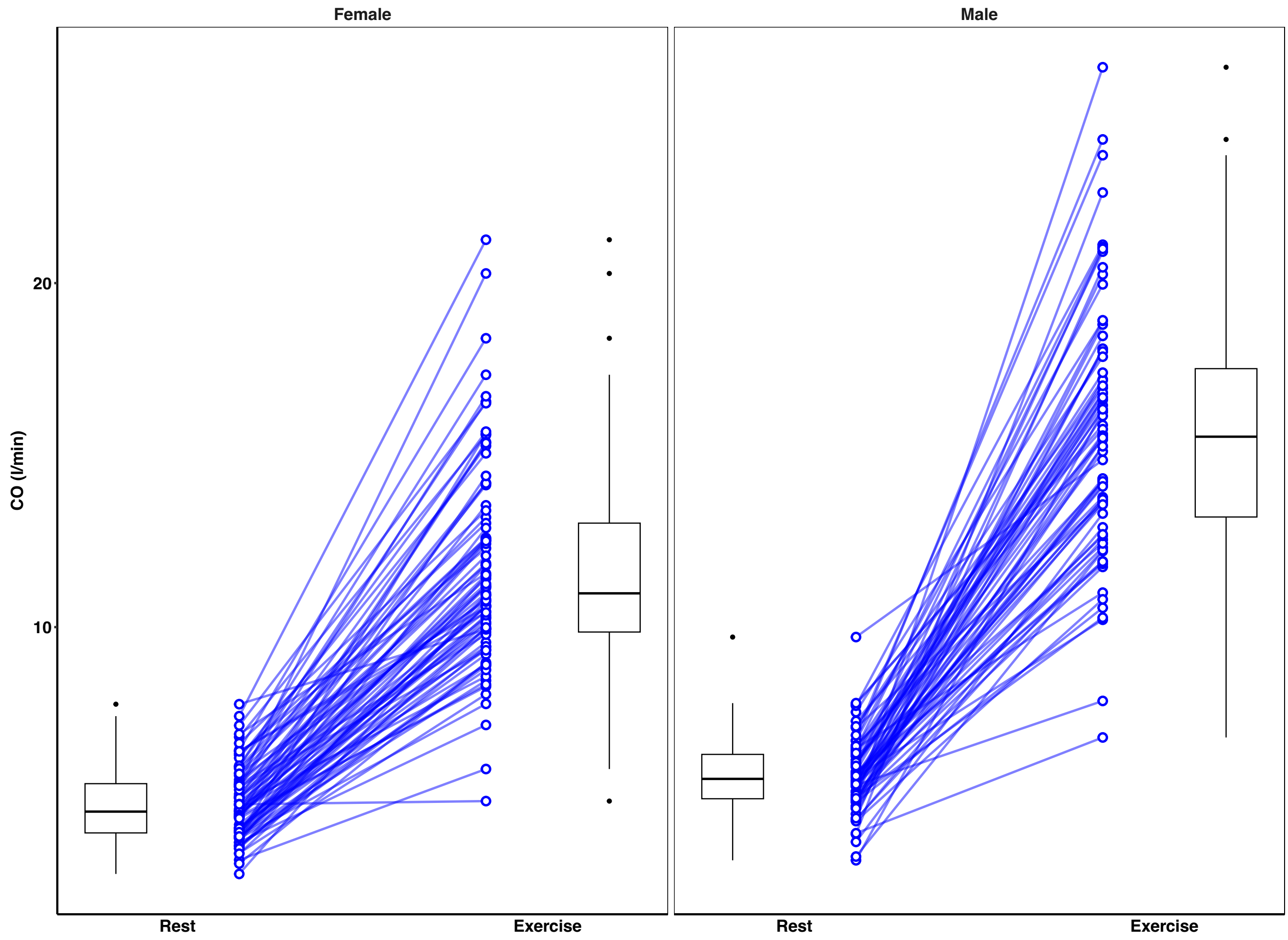

Figure S10. Left ventricular parameters at rest and exercise stratified by gender. Lines join corresponding values. Boxes show median and inter-quartile range (IQR), and whiskers 1.5*IQR. Individual direction of response shown in blue (same) or red (different) with respect to average change. CO, cardiac output. n = 161.

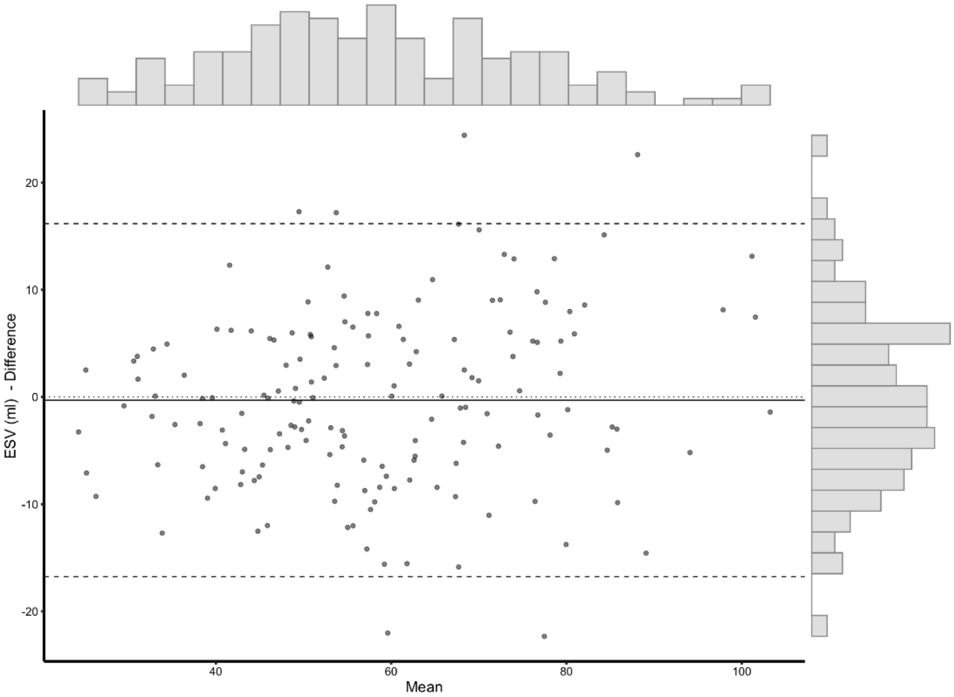

Figure S11. Agreement between methods to assess resting function. Bland-Altman plot with marginal histograms comparing left ventricular parameters derived from retrospectively gated cine images to real time cine images at rest. RT, real time; ESV, end-systolic volume. n = 161.

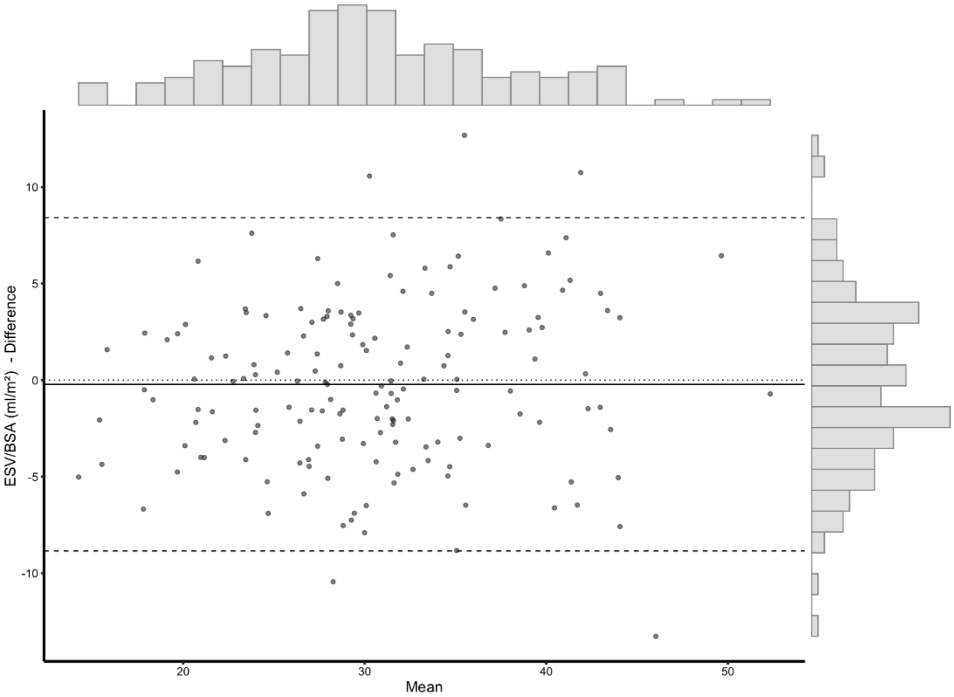

Figure S12. Agreement between methods to assess resting function. Bland-Altman plot with marginal histograms comparing left ventricular parameters derived from retrospectively gated cine images to real time cine images at rest. RT, real time; ESV, end-systolic volume; BSA, body surface area. n = 161.

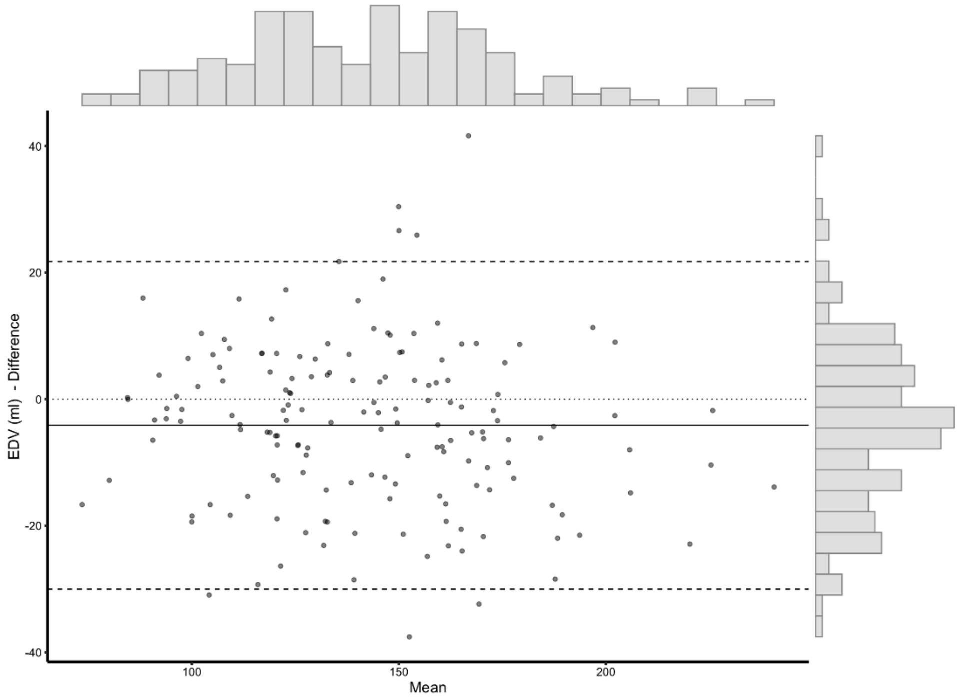

Figure S13. Agreement between methods to assess resting function. Bland-Altman plot with marginal histograms comparing left ventricular parameters derived from retrospectively gated cine images to real time cine images at rest. RT, real time; EDV, end-diastolic volume. n = 161.

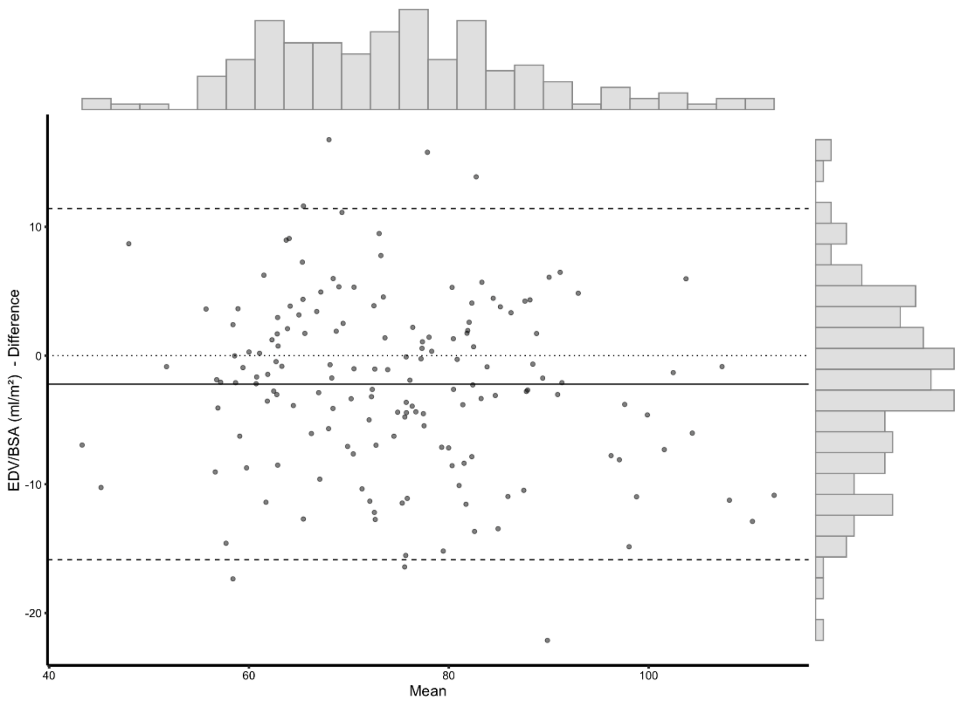

Figure S14. Agreement between methods to assess resting function. Bland-Altman plot with marginal histograms comparing left ventricular parameters derived from retrospectively gated cine images to real time cine images at rest. RT, real time; EDV, end-diastolic volume; BSA, body surface area. n = 161.

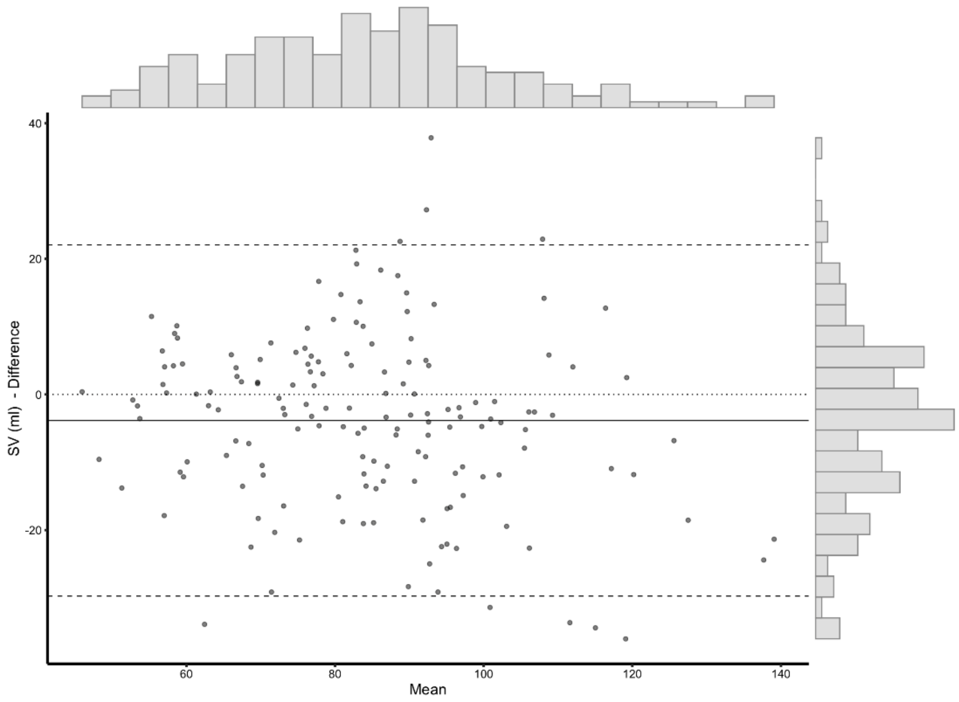

Figure S15. Agreement between methods to assess resting function. Bland-Altman plot with marginal histograms comparing left ventricular parameters derived from retrospectively gated cine images to real time cine images at rest. RT, real time; SV, stroke volume. n = 161.

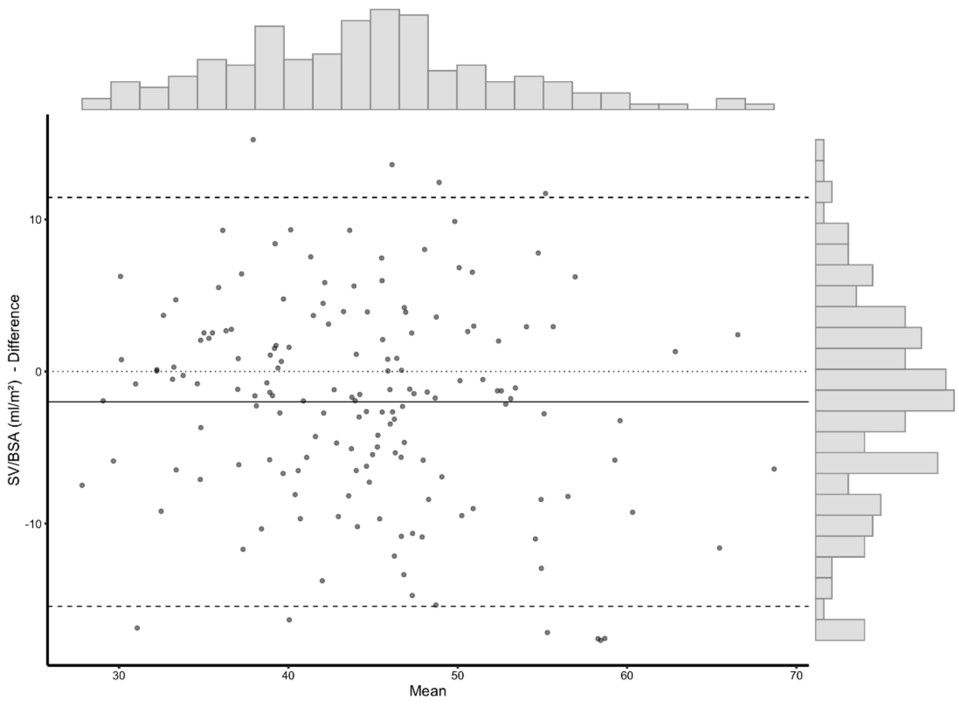

Figure S16. Agreement between methods to assess resting function. Bland-Altman plot with marginal histograms comparing left ventricular parameters derived from retrospectively gated cine images to real time cine images at rest. RT, real time; SV, stroke volume; BSA, body surface area. n = 161.

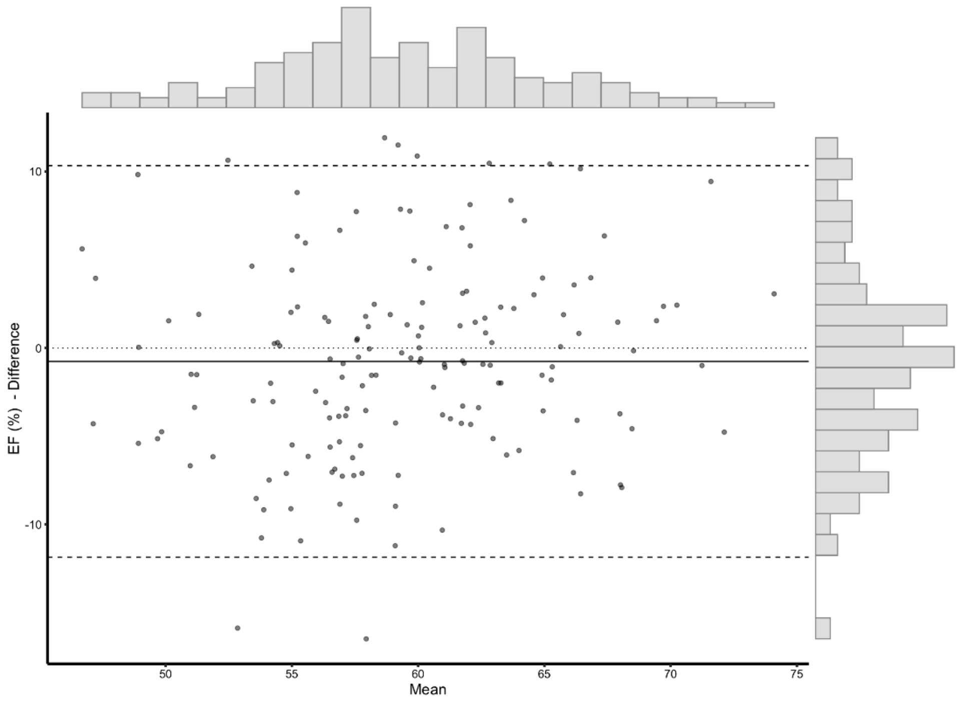

Figure S17. Agreement between methods to assess resting function. Bland-Altman plot with marginal histograms comparing left ventricular parameters derived from retrospectively gated cine images to real time cine images at rest. RT, real time; EF, ejection fraction. n = 161.

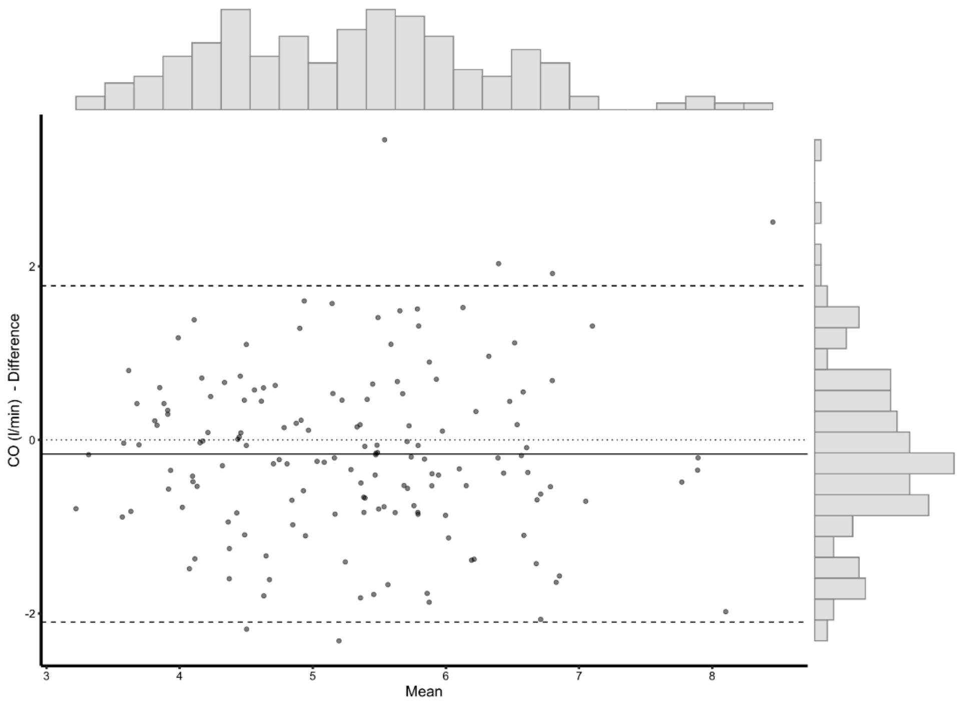

Figure S18. Agreement between methods to assess resting function. Bland-Altman plot with marginal histograms comparing left ventricular parameters derived from retrospectively gated cine images to real time cine images at rest. RT, real time; CO, cardiac output. n = 161.

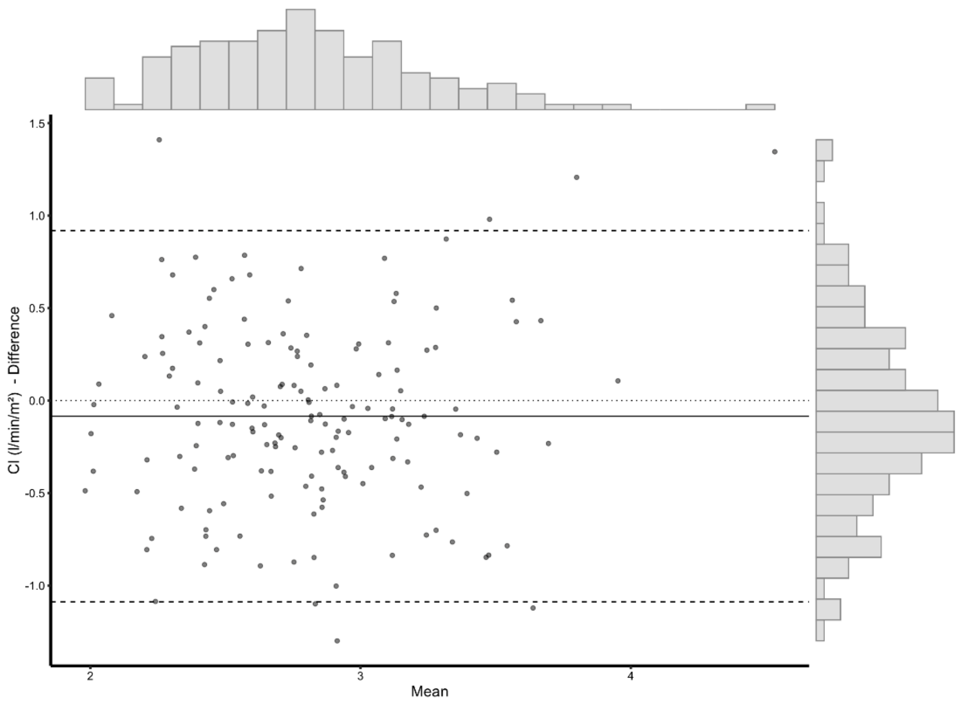

Figure S19. Agreement between methods to assess resting function. Bland-Altman plot with marginal histograms comparing left ventricular parameters derived from retrospectively gated cine images to real time cine images at rest. RT, real time; CI cardiac index. n = 161.

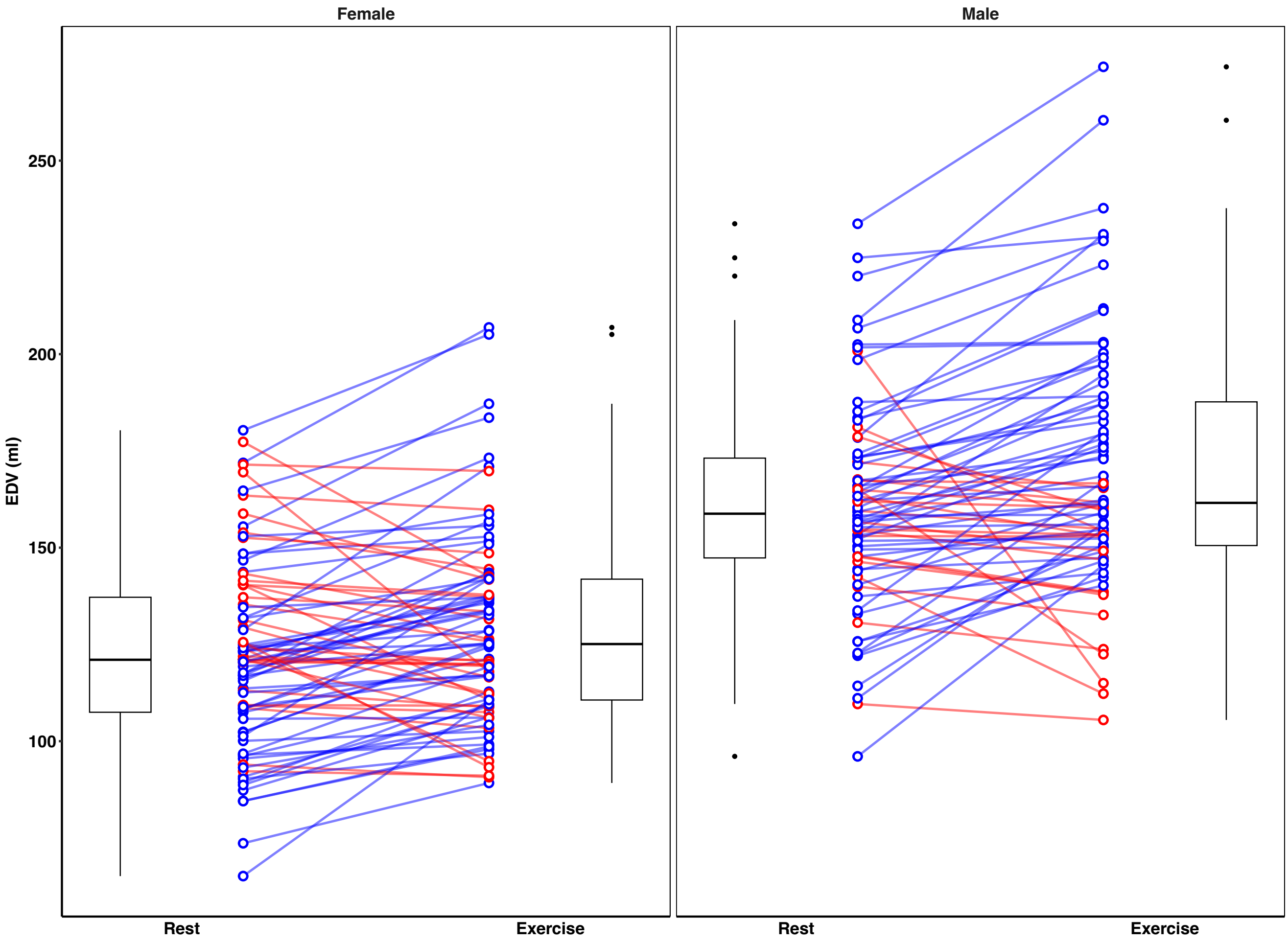

Figure S20. Left ventricular parameters at rest and exercise stratified by gender. Lines join corresponding values. Boxes show median and inter-quartile range (IQR), and whiskers 1.5*IQR. Individual direction of response shown in blue (same) or red (different) with respect to average change. EDV, end-diastolic volume. n = 161.

Supplementary Videos

Video S1. Exercise being performed using a MR-conditional variable resistance supine ergometer**.**

Video S2. Endocardial and epicardial segmentation of the heart on a mid-ventricular real time short axis cine.

Video S3. Stack of real time cines images in the left ventricular short axis.

Supplementary Tables

	Rest				Exercise			
	Overall n = 161	Men n = 76	Women n = 85	P value	Overall n = 161	Men n = 76	Women n = 85	P value
HR (bpm)	64 ± 9	64 ± 11	65 ± 7	0.305	133 ± 19	135 ± 21	130 ± 16	0.116
EDV (ml)	140 ± 32	160 ± 27	122 ± 25	<0.001	148 ± 35	170 ± 32	129 ± 25	<0.001
EDV/BSA (ml/m^2)	74 ± 13	79 ± 12	70 ± 13	<0.001	78 ± 14	83 ± 14	73 ± 12	<0.001
ESV (ml)	58 ± 18	70 ± 16	47 ± 12	<0.001	46 ± 15	54 ± 15	39 ± 12	<0.001
ESV/BSA (ml/m^2)	30 ± 8	34 ± 7	27 ± 7	<0.001	24 ± 7	26 ± 7	22 ± 7	<0.001
SV (ml)	82 ± 18	90 ± 16	75 ± 16	<0.001	102 ± 25	116 ± 24	89 ± 18	<0.001
SV/BSA (ml/m^2)	43 ± 8	44 ± 8	43 ± 9	0.305	54 ± 10	57 ± 11	51 ± 9	<0.001
EF (%)	59 ± 6	56 ± 6	61 ± 6	<0.001	69 ± 7	68 ± 7	70 ± 7	0.104
CO (l/min)	5.2 ± 1.1	5.7 ± 1.1	4.8 ± 1.1	<0.001	13.5 ± 3.9	15.6 ± 3.7	11.7 ± 3.0	<0.001
CI (l/min/m^2)	2.8 ± 0.5	2.8 ± 0.5	2.8 ± 0.5	0.969	7.1 ± 1.7	7.7 ± 1.7	6.6 ± 1.6	<0.001

Table S1. Absolute left ventricular parameters at rest and exercise in men and women. EDV, end-diastolic volume; ESV, end-systolic volume; SV, stroke volume; EF, ejection fraction; CO, cardiac output; CI, cardiac index; BSA, body surface area.

	Overall n = 161	Men n = 76	Women n = 85	P value
HR (bpm)	68 ± 20	72 ± 22	65 ± 18	<0.001
EDV (ml)	8 ± 20	10 ± 22	6 ± 18	0.149
EDV/BSA (ml/m²)	4 ± 10	5 ± 11	4 ± 10	0.323
ESV (ml)	-12 ± 12	-16 ± 13	-8 ± 9	<0.001
ESV/BSA (ml/m²)	-6 ± 6	-8 ± 7	-4 ± 5	0.001
SV (ml)	20 ± 19	26 ± 20	14 ± 18	<0.001
SV/BSA (ml/m²)	10 ± 10	13 ± 9	8 ± 10	0.005
EF (%)	10 ± 8	12 ± 7	8 ± 8	0.005
CO (l/min)	8.3 ± 3.5	10.0 ± 3.6	6.8 ± 2.6	<0.001
CI (l/min/m²)	4.4 ± 1.7	4.9 ± 1.8	3.9 ± 1.4	<0.001

Table S2. Change in left ventricular parameters on exercise. EDV, end-diastolic volume; ESV, end-systolic volume; SV, stroke volume; EF, ejection fraction; CO, cardiac output; CI, cardiac index; BSA, body surface area.

						Post-hoc testing		
	Overall n = 76	20 - 39 years n = 26	40 - 59 years n = 32	60 - 79 years n = 18	*P* value	20-39 vs. 40-59	20-39 vs. 60-79	40-59 vs. 60-79
HR at rest (bpm)	64 ± 11	62 ± 8	66 ± 13	62 ± 9	0.765	-	-	-
HR during exercise (bpm)	135 ± 21	150 ± 17	130 ± 20	124 ± 17	<0.001	<0.001	<0.001	0.49
EDV at rest (ml)	160 ± 27	162 ± 21	161 ± 33	158 ± 22	0.981	-	-	-
EDV during exercise (ml)	170 ± 32	161 ± 26	178 ± 38	169 ± 27	0.317	-	-	-
EDV/BSA at rest (ml/m^2)	79 ± 12	81 ± 10	78 ± 14	77 ± 12	0.878	-	-	-
EDV/BSA during exercise (ml/m^2)	83 ± 14	81 ± 12	86 ± 17	82 ± 12	0.539	-	-	-
ESV at rest (ml)	70 ± 16	72 ± 13	71 ± 17	64 ± 15	0.698	-	-	-
ESV during exercise (ml)	54 ± 15	51 ± 11	56 ± 17	54 ± 17	0.612	-	-	-
ESV/BSA at rest (ml/m^2)	34 ± 7	36 ± 7	35 ± 8	31 ± 7	0.637	-	-	-
ESV/BSA during exercise (ml/m2)	26 ± 7	26 ± 6	27 ± 8	26 ± 8	0.745			
SV at rest (ml)	90 ± 16	90 ± 14	89 ± 20	94 ± 14	0.878	-	-	-
SV during exercise (ml)	116 ± 24	110 ± 23	122 ± 27	115 ± 17	0.317	-	-	-
SV/BSA at rest (ml/m^2)	44 ± 8	45 ± 6	43 ± 9	46 ± 8	0.846	-	-	-
SV/BSA during exercise (ml/m^2)	57 ± 11	55 ± 11	59 ± 11	56 ± 9	0.539	-	-	-
EF at rest (%)	56 ± 6	56 ± 5	55 ± 6	60 ± 6	0.321	-	-	-
EF during exercise (%)	68 ± 7	68 ± 8	69 ± 6	69 ± 7	0.85	-	-	-
CO at rest (l/min)	5.7 ± 1.1	5.6 ± 0.9	5.8 ± 1.3	5.7 ± 0.9	0.957	-	-	-
CO during exercise (l/min)	15.6 ± 3.7	16.5 ± 3.8	15.8 ± 4.0	14.2 ± 2.4	0.317	-	-	-
CI at rest (l/min/m^2)	2.8 ± 0.5	2.8 ± 0.4	2.8 ± 0.6	2.8 ± 0.4	0.981	-	-	-
CI during exercise (l/min/m^2)	7.7 ± 1.7	8.2 ± 1.9	7.6 ± 1.7	7.0 ± 1.3	0.306	-	-	-

Table S3. Absolute left ventricular parameters at rest and exercise in men by age group. EDV, end-diastolic volume; ESV, end-systolic volume; SV, stroke volume; EF, ejection fraction; CO, cardiac output; CI, cardiac index; BSA, body surface area; HR, heart rate.

	Overall<br>n = 76	20 - 39 years<br>n = 26	40 - 59 years<br>n = 32	60 - 79 years<br>n = 18	*P* value	Post-hoc testing<br>20-39 vs. 40-59	20-39 vs. 60-79	40-59 vs. 60-79
HR (bpm)	72 ± 22	88 ± 16	64 ± 22	62 ± 17	<0.001	<0.001	<0.001	0.932
EDV (ml)	10 ± 22	0 ± 24	18 ± 18	11 ± 21	0.033	0.005	0.194	0.51
EDV/BSA (ml/m^2)	5 ± 11	0 ± 12	9 ± 9	5 ± 10	0.035	0.007	0.26	0.487
ESV (ml)	-16 ± 13	-21 ± 13	-15 ± 12	-11 ± 14	0.066	-	-	-
ESV/BSA (ml/m^2)	-8 ± 7	-10 ± 6	-7 ± 6	-5 ± 8	0.066	-	-	-
SV (ml)	26 ± 20	20 ± 21	33 ± 19	22 ± 15	0.059	-	-	-
SV/BSA (ml/m^2)	13 ± 9	10 ± 11	16 ± 8	10 ± 7	0.066	-	-	-
EF (%)	12 ± 7	12 ± 8	13 ± 6	9 ± 7	0.181	-	-	-
CO (l/min)	10.0 ± 3.6	10.9 ± 3.7	10.0 ± 4.0	8.5 ± 2.0	0.102	-	-	-
CI (l/min/m^2)	4.9 ± 1.8	5.5 ± 1.9	4.8 ± 1.8	4.2 ± 1.1	0.073	-	-	-

Table S4. Change in left ventricular parameters on exercise in men by age group. EDV, end-diastolic volume; ESV, end-systolic volume; SV, stroke volume; EF, ejection fraction; CO, cardiac output; CI, cardiac index; BSA, body surface area; HR, heart rate.

	Overall n = 85	20 - 39 years n = 34	40 - 59 years n = 35	60 - 79 years n = 26	*P* value	Post-hoc testing 20-39 vs. 40-59	20-39 vs. 60-79	40-59 vs. 60-79
HR at rest (bpm)	65 ± 7	66 ± 8	65 ± 7	65 ± 7	0.707	-	-	-
HR during exercise (bpm)	130 ± 16	140 ± 17	131 ± 13	121 ± 14	<0.001	0.041	<0.001	0.039
EDV at rest (ml)	122 ± 25	133 ± 26	122 ± 22	113 ± 23	0.063	-	-	-
EDV during exercise (ml)	129 ± 25	138 ± 32	126 ± 21	122 ± 18	0.111	-	-	-
EDV/BSA at rest (ml/m^2)	70 ± 13	75 ± 14	68 ± 11	67 ± 13	0.070	-	-	-
EDV/BSA during exercise (ml/m^2)	73 ± 12	78 ± 16	71 ± 11	73 ± 10	0.118	-	-	-
ESV at rest (ml)	47 ± 12	53 ± 14	46 ± 10	43 ± 11	0.063	-	-	-
ESV during exercise (ml)	39 ± 12	41 ± 14	38 ± 13	39 ± 10	0.742	-	-	-
ESV/BSA at rest (ml/m^2)	27 ± 7	30 ± 7	26 ± 6	26 ± 6	0.063	-	-	-
ESV/BSA during exercise (ml/m^2)	22 ± 7	23 ± 8	22 ± 7	23 ± 6	0.622	-	-	-
SV at rest (ml)	75 ± 16	80 ± 15	75 ± 17	70 ± 16	0.134	-	-	-
SV during exercise (ml)	89 ± 18	97 ± 24	88 ± 15	83 ± 15	0.067	-	-	-
SV/BSA at rest (ml/m^2)	43 ± 9	45 ± 8	42 ± 8	41 ± 9	0.317	-	-	-
SV/BSA during exercise (ml/m^2)	51 ± 9	55 ± 12	49 ± 7	49 ± 7	0.079	-	-	-
EF at rest (%)	61 ± 6	60 ± 6	62 ± 7	62 ± 6	0.637	-	-	-
EF during exercise (%)	70 ± 7	70 ± 7	70 ± 7	68 ± 7	0.566	-	-	-
CO at rest (l/min)	4.8 ± 1.1	5.2 ± 1.1	4.8 ± 1.0	4.5 ± 1.0	0.070	-	-	-
CO during exercise (l/min)	11.7 ± 3.0	13.6 ± 3.6	11.5 ± 2.2	10.1 ± 2.2	<0.001	0.011	<0.001	0.143
CI at rest (l/min/m^2)	2.8 ± 0.5	3.0 ± 0.6	2.7 ± 0.5	2.6 ± 0.5	0.108	-	-	-
CI during exercise (l/min/m^2)	6.6 ± 1.6	7.7 ± 2.0	6.4 ± 1.2	6.0 ± 1.2	<0.001	0.005	<0.001	0.49

Table S5. Absolute left ventricular parameters at rest and exercise in women by age group. EDV, end-diastolic volume; ESV, end-systolic volume; SV, stroke volume; EF, ejection fraction; CO, cardiac output; CI, cardiac index; BSA, body surface area; HR, heart rate.

	Overall n = 85	20 - 39 years n = 34	40 - 59 years n = 35	60 - 79 years n = 26	*P* value	Post-hoc testing 20-39 vs. 40-59	20-39 vs. 60-79	40-59 vs. 60-79
HR (bpm)	65 ± 18	74 ± 18	66 ± 15	57 ± 17	0.006	0.158	0.001	0.09
EDV (ml)	6 ± 18	5 ± 15	5 ± 20	10 ± 17	0.6	-	-	-
EDV/BSA (ml/m^2)	4 ± 10	3 ± 8	3 ± 11	6 ± 10	0.6	-	-	-
ESV (ml)	-8 ± 9	-12 ± 10	-8 ± 9	-4 ± 8	0.012	0.112	0.004	0.284
ESV/BSA (ml/m^2)	-4 ± 5	-7 ± 6	-4 ± 5	-2 ± 5	0.012	0.098	0.005	0.334
SV (ml)	14 ± 18	17 ± 15	13 ± 19	14 ± 18	0.6	-	-	-
SV/BSA (ml/m^2)	8 ± 10	10 ± 8	7 ± 10	8 ± 11	0.6	-	-	-
EF (%)	8 ± 8	10 ± 7	8 ± 8	6 ± 8	0.376	-	-	-
CO (l/min)	6.8 ± 2.6	8.3 ± 3.1	6.6 ± 2.1	5.7 ± 1.9	0.006	0.022	<0.001	0.282
CI (l/min/m^2)	3.9 ± 1.4	4.7 ± 1.7	3.7 ± 1.2	3.4 ± 1.1	0.006	0.018	0.001	0.519

Table S6. Change in left ventricular parameters on exercise in women by age group. EDV, end-diastolic volume; ESV, end-systolic volume; SV, stroke volume; EF, ejection fraction; CO, cardiac output; CI, cardiac index; BSA, body surface area; HR, heart rate.

	Intra-Observer ICC	Inter-Observer ICC	Agreement between Methods at Rest
	n = 50	n = 50	n = 161
EDV at rest	0.98	0.99	4 ± 25
EDV during exercise	0.97	0.97	[redacted]
EDV/BSA at rest	0.97	0.98	2 ± 8
EDV/BSA during exercise	0.95	0.95	[redacted]
ESV at rest	0.97	0.95	0 ± 16
ESV during exercise	0.98	0.98	[redacted]
ESV/BSA at rest	0.97	0.95	0 ± 8
ESV/BSA during exercise	0.98	0.98	[redacted]
SV at rest	0.95	0.92	4 ± 25
SV during exercise	0.93	0.93	[redacted]
SV/BSA at rest	0.94	0.90	2 ± 13
SV/BSA during exercise	0.89	0.89	[redacted]
EF at rest	0.92	0.83	1 ± 11
EF during exercise	0.94	0.92	[redacted]
CO at rest	0.96	0.93	0.2 ± 1.9
CO during exercise	0.96	0.95	[redacted]
CI at rest	0.94	0.90	0.1 ± 1
CI during exercise	0.95	0.94	[redacted]

Table S7. Intra- and inter-observer variability. Mean difference and lines of agreement between retrospectively gated cine images to real time cine images at rest. EDV, end-diastolic volume; ESV, end-systolic volume; SV, stroke volume; EF, ejection fraction; CO, cardiac output; CI, cardiac index; BSA, body surface area.

	Sedentary<br><br>n = 3	Lightly Active<br><br>n = 28	Moderately Active<br>n = 33	Very Active<br><br>n = 12	*P* value
HR at rest (bpm)	71 ± 6	65 ± 9	63 ± 11	58 ± 11	0.297
HR during exercise (bpm)	139 ± 7	138 ± 19	132 ± 23	140 ± 21	0.849
EDV at rest (ml)	142 ± 34	151 ± 22	166 ± 25	172 ± 29	0. 316
EDV during exercise (ml)	153 ± 6	166 ± 26	170 ± 34	184 ± 39	0.404
EDV/BSA at rest (ml/m2)	64 ± 10	74 ± 11	81 ± 11	86 ± 13	0.035
EDV/BSA during exercise (ml/m2)	70 ± 6	81 ± 10	83 ± 14	93 ± 19	0.229
ESV at rest (ml)	69 ± 13	64 ± 13	72 ± 14	78 ± 20	0.316
ESV during exercise (ml)	47 ± 8	49 ± 13	57 ± 17	60 ± 13	0.229
ESV/BSA at rest (ml/m2)	31 ± 3	31 ± 6	35 ± 7	39 ± 9	0.134
ESV/BSA during exercise (ml/m2)	22 ± 7	24 ± 6	28 ± 7	30 ± 6	0.229
SV at rest (ml)	73 ± 21	87 ± 14	94 ± 17	94 ± 11	0.316
SV during exercise (ml)	106 ± 14	118 ± 20	113 ± 25	124 ± 31	0.422
SV/BSA at rest (ml/m2)	33 ± 7	42 ± 7	46 ± 7	47 ± 5	0.035
SV/BSA during exercise (ml/m2)	48 ± 3	57 ± 8	56 ± 11	62 ± 15	0.229
EF at rest (%)	51 ± 3	58 ± 6	57 ± 6	55 ± 4	0.316
EF during exercise (%)	69. ± 7	71 ± 6	67 ± 7	67 ± 7	0.235
CO at rest (l/min)	5.1 ± 1.6	5.6 ± 1.0	5.9 ± 1.2	5.4 ± 0.7	0.463
CO during exercise (l/min)	14.8 ± 2.3	16.1 ± 2.9	14.9 ± 3.7	17.1 ± 4.5	0.229
CI at rest (l/min/m2)	2.3 ± 0.5	2.7 ± 0.3	2.9 ± 0.6	2.7 ± 0.3	0.316
CI during exercise (l/min/m2)	6.7 ± 0.7	7.8 ± 1.4	7.3 ± 1.8	8.5 ± 2.0	0.229

Table S8. Absolute left ventricular parameters at rest and exercise in men by activity level. EDV, end-diastolic volume; ESV, end-systolic volume; SV, stroke volume; EF, ejection fraction; CO, cardiac output; CI, cardiac index; BSA, body surface area; HR, heart rate.

	Sedentary<br><br>n = 9	Lightly Active<br><br>n = 37	Moderately Active<br>n = 25	Very Active<br><br>n = 14	*P* value
HR at rest (bpm)	70 ± 7	65 ± 7	66 ± 6	62 ± 7	0.273
HR during exercise (bpm)	131 ± 17	129 ± 18	131 ± 10	132 ± 18	0.989
EDV at rest (ml)	121 ± 27	117 ± 20	122 ± 20	138 ± 32	0.363
EDV during exercise (ml)	125 ± 25	124 ± 20	129 ± 21	143 ± 34	0.755
EDV/BSA at rest (ml/m2)	64 ± 8	68 ± 11	68 ± 10	80 ± 16	0.194
EDV/BSA during exercise (ml/m2)	66 ± 8	72 ± 11	72 ± 11	82 ± 16	0.308
ESV at rest (ml)	45 ± 14	45 ± 9	46 ± 11	58 ± 14	0.085
ESV during exercise (ml)	36 ± 15	37 ± 10	40 ± 10	48 ± 15	0.308
ESV/BSA at rest (ml/m2)	23 ± 5	26 ± 5	26 ± 6	33 ± 7	0.024
ESV/BSA during exercise (ml/m2)	19 ± 6	21 ± 5	23 ± 6	28 ± 8	0.230
SV at rest (ml)	77 ± 16	72 ± 14	75 ± 16	80 ± 21	0.695
SV during exercise (ml)	89 ± 14	87 ± 15	89 ± 18	95 ± 27	0.989
SV/BSA at rest (ml/m2)	40 ± 5	42 ± 8	42 ± 7	46 ± 11	0.695
SV/BSA during exercise (ml/m2)	47 ± 6	51 ± 8	50 ± 7	54 ± 13	0.755
EF at rest (%)	64 ± 6	62 ± 5	62 ± 7	58 ± 5	0.273
EF during exercise (%)	72 ± 7	71 ± 6	69 ± 6	66 ± 8	0.446
CO at rest (l/min)	5.3 ± 1.0	4.6 ± 0.9	4.9 ± 1.2	4.9 ± 1.2	0.594
CO during exercise (l/min)	11.7 ± 2.7	11.2 ± 2.4	11.6 ± 2.4	12.8 ± 4.7	0.989
CI at rest (l/min/m2)	2.8 ± 0.4	2.7 ± 0.5	2.8 ± 0.5	2.8 ± 0.6	0.978
CI during exercise (l/min/m2)	6.3 ± 1.5	6.6 ± 1.4	6.5 ± 1.1	7.3 ± 2.4	0.988

Table S9. Absolute left ventricular parameters at rest and exercise in women by activity level. EDV, end-diastolic volume; ESV, end-systolic volume; SV, stroke volume; EF, ejection fraction; CO, cardiac output; CI, cardiac index; BSA, body surface area; HR, heart rate.

Genetic Sequencing

Genetic sequencing of 169 genes, associated with inherited cardiac conditions, was conducted using the Illumina MiSeq, Illumina NextSeq, or Life Technologies SOLiD 5500xl platforms, after target enrichment using in-solution hybridisation (Illumina Nextera or Agilent SureSelect), as previously described.[1–3] Variants were annotated and filtered using a validated bioinformatics pipeline.[4] Rare variants were defined by a minor allele frequency of <10-4 from population data in gnomAD applying stringent filtering. Individuals harbouring variants that would be called pathogenic or likely pathogenic if identified in a patient with an inherited cardiac condition were considered genotype-positive.

Supplementary References

1. Roberts AM, Ware JS, Herman DS, Schafer S, Baksi J, Bick AG, Buchan RJ, Walsh R, John S, Wilkinson S, Mazzarotto F, Felkin LE, Gong S, MacArthur JAL, Cunningham F, Flannick J, Gabriel SB, Altshuler DM, Macdonald PS, Heinig M, Keogh AM, Hayward CS, Banner NR, Pennell DJ, O'Regan DP, San TR, Marvao A de, Dawes TJW, Gulati A, Birks EJ, Yacoub MH, Radke M, Gotthardt M, Wilson JG, O'Donnell CJ, Prasad SK, Barton PJR, Fatkin D, Hubner N, Seidman JG, Seidman CE, and Cook SA. Integrated allelic, transcriptional, and phenomic dissection of the cardiac effects of titin truncations in health and disease. *Sci Transl Med*. 2015;7:270ra6.
2. Pua CJ, Bhalshankar J, Miao K, Walsh R, John S, Lim SQ, Chow K, Buchan R, Soh BY, Lio PM, Lim J, Schafer S, Lim JQ, Tan P, Whiffin N, Barton PJ, Ware JS, and Cook SA. Development of a comprehensive sequencing assay for inherited cardiac condition genes. *J Cardiovasc Transl Res*. 2016;9:3–11.
3. Schafer S, Marvao A de, Adami E, Fiedler LR, Ng B, Khin E, Rackham OJ, Heesch S van, Pua CJ, Kui M, Walsh R, Tayal U, Prasad SK, Dawes TJ, Ko NS, Sim D, Chan LL, Chin CW, Mazzarotto F, Barton PJ, Kreuchwig F, Kleijn DP de, Totman T, Biffi C, Tee N, Rueckert D, Schneider V, Faber A, Regitz-Zagrosek V, Seidman JG, Seidman CE, Linke WA, Kovalik JP, O'Regan D, Ware JS, Hubner N, and Cook SA. Titin-truncating variants affect heart function in disease cohorts and the general population. *Nat Genet*.

2017;49:46–53.

4. Lota AS, Hazebroek MR, Theotokis P, Wassall R, Salmi S, Halliday BP, Tayal U, Verdonschot J, Meena D, Owen R, et al. Genetic architecture of acute myocarditis and the overlap with inherited cardiomyopathy. *Circulation*. 2022;146:1123–34.
